# FOXF2 reprograms breast cancer cells into bone metastasis seeds

**DOI:** 10.1038/s41467-019-10379-7

**Published:** 2019-06-20

**Authors:** Shuo Wang, Gui-Xi Li, Cong-Cong Tan, Rui He, Li-Juan Kang, Jun-Tao Lu, Xiao-Qing Li, Qing-Shan Wang, Pei-Fang Liu, Qiong-Li Zhai, Yu-Mei Feng

**Affiliations:** 1 Department of Biochemistry and Molecular Biology, Tianjin Medical University Cancer Institute and Hospital, National Clinical Research Center of Cancer, Tianjin, 300060 China; 2 Key Laboratory of Breast Cancer Prevention and Treatment of the Ministry of Education, Tianjin Medical University Cancer Institute and Hospital, National Clinical Research Center of Cancer, Tianjin, 300060 China; 3 Department of Radiology, Tianjin Medical University Cancer Institute and Hospital, National Clinical Research Center of Cancer, Tianjin, 300060 China; 4 Department of Pathology, Tianjin Medical University Cancer Institute and Hospital, National Clinical Research Center of Cancer, Tianjin, 300060 China

**Keywords:** Cancer, Oncology

## Abstract

Bone metastases occur in most advanced breast cancer patients and cause serious skeletal-related complications. The mechanisms by which bone metastasis seeds develop in primary tumors and specifically colonize the bone remain to be elucidated. Here, we show that forkhead box F2 (FOXF2) functions as a master transcription factor for reprogramming cancer cells into an osteomimetic phenotype by pleiotropic transactivation of the BMP4/SMAD1 signaling pathway and bone-related genes that are expressed at early stages of bone differentiation. The epithelial-to-osteomimicry transition regulated by FOXF2 confers a tendency on cancer cells to metastasize to bone which leads to osteolytic bone lesions. The BMP antagonist Noggin significantly inhibits FOXF2-driven osteolytic bone metastasis of breast cancer cells. Thus, targeting the FOXF2-BMP/SMAD axis might be a promising therapeutic strategy to manage bone metastasis. The role of FOXF2 in transactivating bone-related genes implies a biological function of FOXF2 in regulating bone development and remodeling.

## Abstract (web summary)

Bone metastasis in breast cancer patients causes major skeletal-related complications. Here, the authors show that FOXF2/BMP/SMAD pathway plays a major role in bone metastasis and suggest targeting this axis to manage bone metastasis.

## Introduction

Bone is the most common site of distant metastasis in breast cancer patients. Bone metastasis occurs in 80% of patients with advanced breast cancer. Most breast cancer bone metastases generate osteolytic bone lesions associated with a variety of bone complications that seriously affect the life quality of patients, and are associated with a dismal prognosis^1^. The prevention and treatment of bone metastasis remain challenging in the clinic. Elucidating the cellular and molecular mechanisms underlying breast cancer osteotropism could contribute to the development of strategies for predicting and managing bone metastasis. Organ-specific metastasis depends on the intrinsic molecular characteristics of cancer cells, referred to as “pre-selected” seeds^2^, and a suitable microenvironment in host organs prepared by signals released from primary and/or disseminated cancer cells, referred to as a “pre-metastatic niche”^3^. However, the mechanisms by which primary cancer cells are programmed into bone metastasis seeds and disseminated cancer cells interact with the skeletal microenvironment to flourish in bone and generate osteolytic bone lesions remain to be elucidated.

Breast cancer cells can obtain osteomimetic features to resemble osteocytes by ectopically coexpressing bone-related genes (BRGs) involved in bone and bone-matrix remodeling. Osteomimetic cancer cells possessing bone metastatic capability^4^ preferentially home to the bone microenvironment^5^, wherein they survive^6^ and colonize^7,8^. Our previous gene expression profiling data set^9^ and online data sets^10^ of primary breast cancer tissues indicate that the gene encoding mesenchymal transcription factor (TF) forkhead box F2 (FOXF2) is ectopically coexpressed with BRGs. Among the set of BRGs, multiple genes are candidate transcriptional targets of FOXF2. FOXF2 normally plays critical roles in the maintenance of tissue homeostasis by promoting the differentiation of mesenchymal cells and inhibiting the mesenchymal transformation of adjacent epithelial cells during embryonic development and tissue differentiation^11^. Studies by our group^12–15^ and other groups^16–19^ have demonstrated that the deregulation of FOXF2 expression is involved in the tumorigenesis, progression, and metastasis of breast cancer and other cancer types. FOXF2 deficiency significantly accelerates the visceral metastasis of basal-like breast cancer (BLBC)^12^. However, the role of high ectopic FOXF2 expression in breast cancer cells remains to be explored.

Here, we provide clinical and experimental evidence to illustrate the role of FOXF2 in breast cancer bone metastasis and uncover mechanisms underlying the osteomimetic formation and osteolytic bone metastasis of breast cancer cells, in which FOXF2 programs epithelium-to-osteomimicry transition (EOT) by pleiotropic transactivation of the BMP4/SMAD1 signaling pathway and bone-related genes that are expressed at early stages of bone differentiation. Our findings suggest that targeting the FOXF2-BMP/SMAD axis might be a promising therapeutic strategy to manage breast cancer bone metastasis.

## Results

### *FOXF2* expression is correlated with bone-specific metastasis

**Fig. 1:**
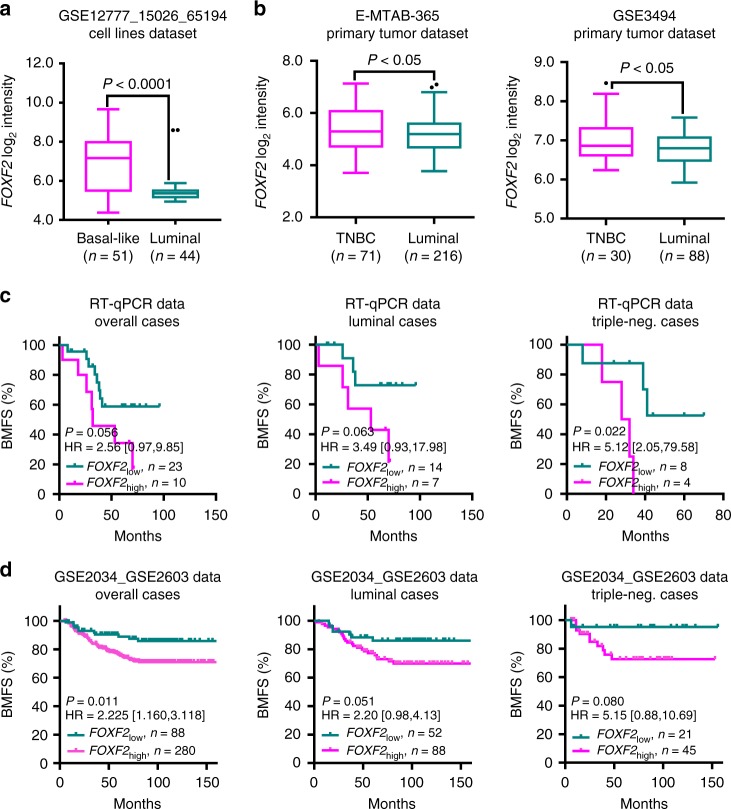
Breast cancers with high *FOXF2* expression have a propensity to metastasize to bone. **a–b** *FOXF2* expression levels in the luminal and basal-like/triple-negative subtypes of human breast cancer were compared by chi-square tests. *FOXF2* mRNA levels were mined from the GSE12777_GSE15026_GSE65194 data set of breast cancer cell lines (*n* = 95); **a** and the E-MTAB-365 (*n* = 287) and GSE3494 (*n* = 118) data sets of primary breast cancer tissues (**b**). The boxplots show the minimum, 25 percentile, median, 75 percentile, maximum, and outliers. **c** *FOXF2* mRNA levels in primary breast cancer tissues that developed distant metastasis (*n* = 33) were detected by RT-qPCR. **d** *FOXF2* mRNA levels in primary breast cancer tissues (*n* = 368) were mined from the GSE2034_GSE2603 data set. The Kaplan–Meier survival curve shows the BMFS status of patients in the *FOXF2*_high_ and *FOXF2*_low_ groups. The overall, luminal subtype and TNBC subtype cases were analyzed separately

To investigate the role of FOXF2 in breast cancer bone metastasis, we first analyzed the *FOXF2* expression pattern in the luminal and triple-negative/basal-like subtypes of breast cancer based on the GSE12777_GSE15026_GSE65194 data set of human breast cancer cell lines and the E-MTAB-365 and GSE3494 data sets of primary breast cancer tissues. The results confirmed our previously published result that *FOXF2* mRNA levels were significantly higher in triple-negative/basal-like subtype than in luminal subtype in both cell lines (Fig. 1a) and tissues (Fig. 1b). Then, we analyzed the relationship between *FOXF2* expression and organ specificity of metastasis in the luminal and triple-negative subtypes of breast cancer. *FOXF2* mRNA levels in primary breast cancer tissues that developed distant metastasis were detected by reverse transcription–quantitative polymerase chain reaction (RT-qPCR). The patients were divided into high *FOXF2* mRNA level (*FOXF2*_high_) and low *FOXF2* mRNA level (*FOXF2*_low_) groups, using the optimal cutoff value of *FOXF2* mRNA expression for distinguishing bone metastasis-free survival (BMFS) statuses in overall cases and cases stratified by subtypes. Kaplan–Meier survival analysis showed that bone metastasis was a more frequent occurrence in patients in *FOXF2*_high_ group than in those in *FOXF2*_low_ group. Stratified analysis based on the hormone receptor status of tumors revealed that *FOXF2*_high_ patients in both luminal and triple-negative breast cancer (TNBC) groups had a shorter BMFS (Fig. 1c). The validation based on the GSE2034_GSE2603 data set confirmed that patients with *FOXF2*_high_ tumors had a shorter BMFS than those with *FOXF2*_low_ tumors among overall cases and among stratified cases of luminal and TNBC subtypes (Fig. 1d). We also analyzed the relationship between *FOXF2* expression and distant metastasis-free survival (DMFS) or non-bone/other organ metastasis-free survival (NBMFS) in overall cases and in different subtype cases based on our RT-qPCR data of primary breast cancer tissues. The results showed that *FOXF2* mRNA level was positively correlated with DMFS in TNBC subtype and with NBMFS in both luminal and TNBC subtypes (Supplementary Fig. 1). These data indicate that breast cancer with high FOXF2 expression has a propensity to metastasize to bone, which is not affected by hormone receptor status.

### FOXF2 drives bone-specific breast cancer metastasis

**Fig. 2:**
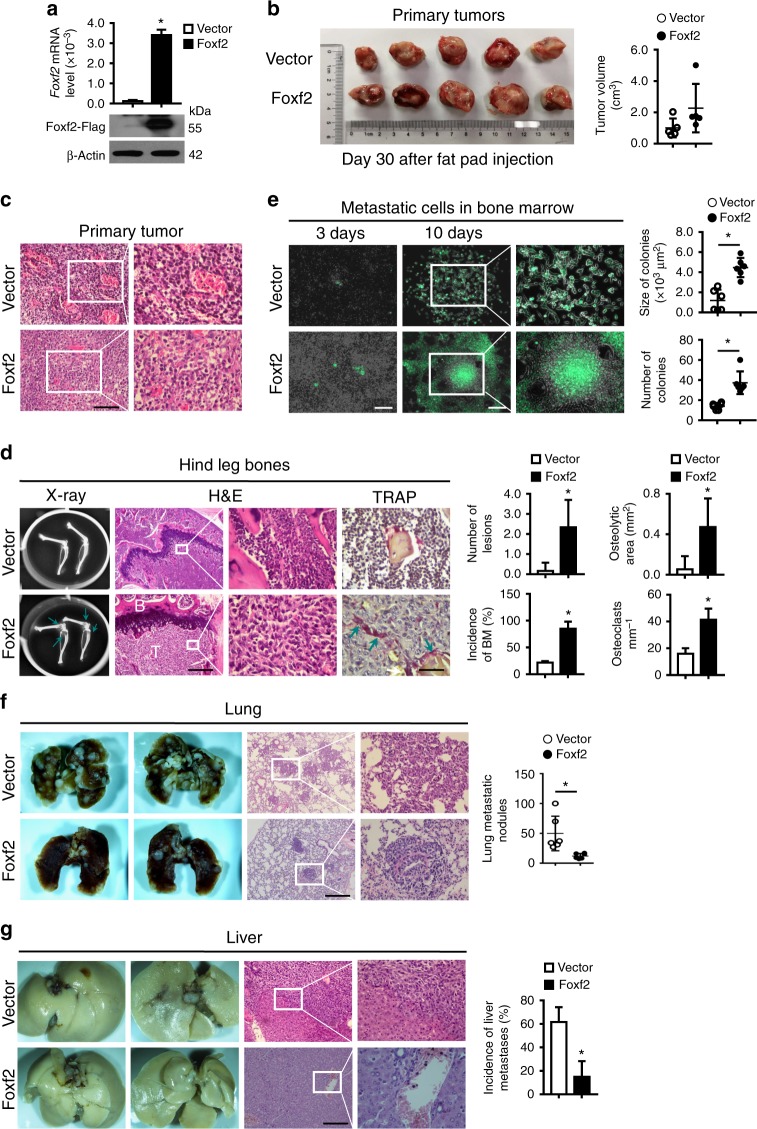
Foxf2 promotes the bone-specific metastasis of breast cancer cells in vivo. A total of 2 × 10^5^ 4T1 cells infected with LV-Foxf2-EGFP (Foxf2) or LV-EGFP vector (Vector) were injected into the fat pad of female nude mice (*n* = 5 per group). The mice were killed by cervical dislocation on day 30 after cell injection. **a** Foxf2 mRNA and protein levels in the indicated cells were detected by RT-qPCR and immunoblot. **b** The volume of xenograft tumor volume was calculated. **c** Histological evaluation of xenograft tumors was identified after H&E staining. Scale bars: 100 μm. **d** The osteolytic lesions and metastases in hind leg bones were observed by X-ray, H&E staining, and TRAP staining. The number of osteolytic lesions, osteolytic area, and log_2_ signal intensity in hind-leg bones detected by X-ray were calculated. Scale bars: 500 μm for H&E staining and 50 μm for TRAP staining. Arrows point to osteolytic lesions or TRAP+ cells. **e** The metastatic cells in the bone marrow of the hind legs were collected and cultured in vitro for 3 or 10 days. The size and the number of metastatic cell colonies were determined under a fluorescence microscope and statistically analyzed. Scale bars, 200 μm. **f**–**g** Metastatic nodules on two sides of the lung (**f**) and liver (**g**) surface were photographed, identified by H&E staining and statistically analyzed. The metastatic nodules were counted, or the incidence of metastasis was calculated. Scale bars: 200 μm. *P < 0.05 by Student’s *t* test. Error bars are defined as s.d.

To investigate the role of FOXF2 in regulating breast cancer bone metastasis, we established 4T1 cells stably overexpressing Foxf2–Flag fusion protein (4T1-Foxf2) and their controls (4T1-Vector) via lentiviral infection (Fig. 2a) for mouse xenograft experiments. The cells were orthotopically injected into the fat pad of female nude mice. The primary tumors formed by 4T1-Foxf2 cells were larger than those formed by 4T1-Vector cells (Fig. 2b), and there was more angiogenesis in 4T1-Vector tumors than in 4T1-Foxf2 tumors (Fig. 2c). X-ray images of hind leg bones showed that 4T1-Foxf2 tumors led to serious osteolytic bone lesions, which were validated by hematoxylin and eosin (H&E) and tartrate-resistant acid phosphatase (TRAP) staining, whereas mice-bearing 4T1-Vector tumors had significantly fewer osteolytic bone lesions (Fig. 2d). The metastatic 4T1-Foxf2 cells isolated from the bone marrow and cultured in vitro formed more colonies, and the colonies were larger than those formed by metastatic 4T1-Vector cells (Fig. 2e). However, Foxf2 overexpression significantly suppressed lung metastases that were observable on the lung surfaces and identified by histological examination (Fig. 2f). Liver metastases were also suppressed by Foxf2 overexpression and identified by histological examination in the livers of mice-bearing 4T1-Vector tumors only (Fig. 2g). We further validated the role of Foxf2 in regulating breast cancer metastasis using a 4T1/BALB/c orthotopic xenograft mouse models with Foxf2 loss- and gain-of-function (Supplementary Fig. 2a–e). The results confirmed the role of FOXF2 in promoting bone metastasis and suppressing lung and liver metastases. The role of FOXF2 in suppressing the visceral metastasis of BLBC is consistent with the findings in our previous report^12^.

### FOXF2 enhances bone-specific metastatic potential

**Fig. 3:**
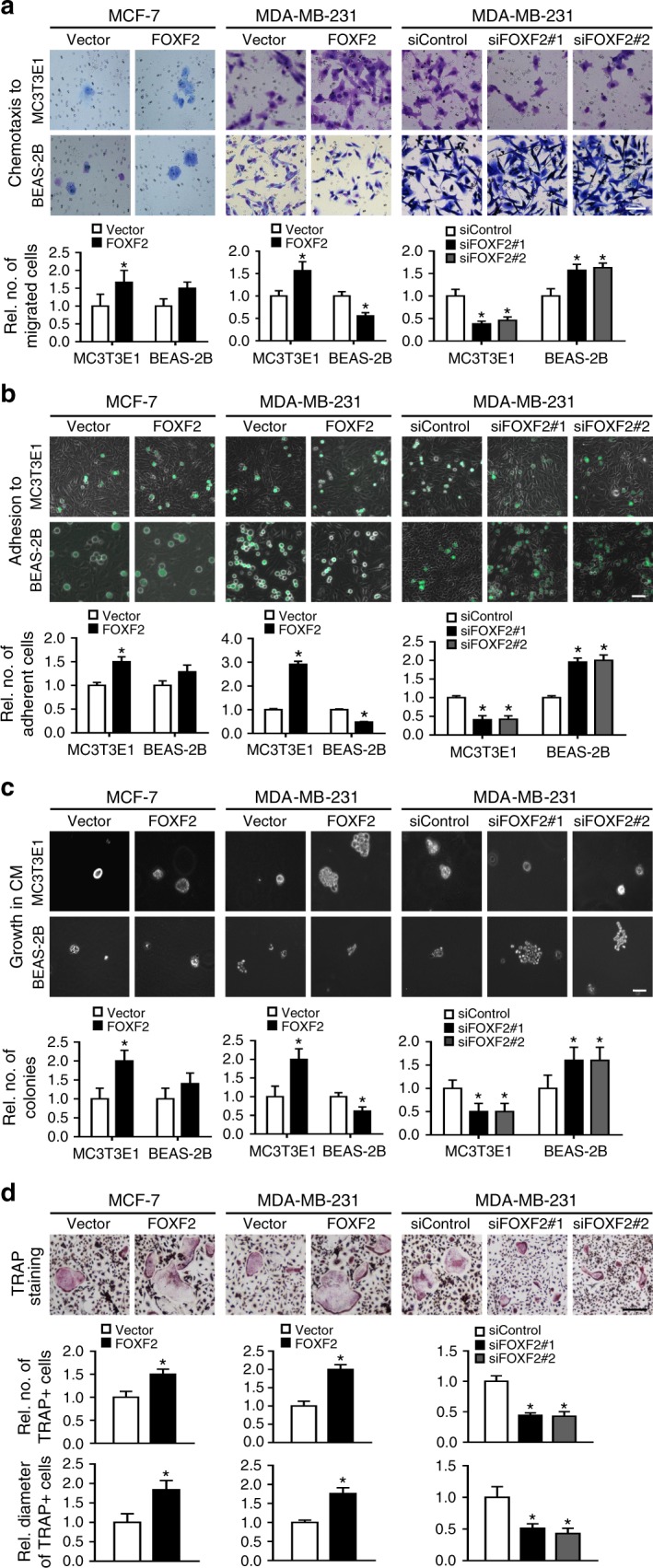
FOXF2 enhances the bone-specific metastasis potential of breast cancer cells. MCF-7 and MDA-MB-231 cells were treated as indicated. **a** Chemotactic migration of cancer cells toward MC3T3E1 or BEAS-2B cells was assessed by transwell assays. **b** Adhesion of cancer cells to MC3T3E1 or BEAS-2B cells was assessed by adding cancer cells to MC3T3E1 or BEAS-2B cells at 100% saturation, followed by incubation for 30 min. **c** Anchorage-independent growth of cancer cells in the CM from MC3T3E1 or BEAS-2B cells was assessed by soft agar colony-formation assays. **d** The ability of cancer cells to induce osteoclast differentiation was assessed by incubation of pre-induced primary preosteoclasts with cancer cell CM containing 50 ng ml^–1^ RANKL. The number and size of mature osteoclasts with TRAP-positive multinucleated (≥3 nuclei) were analyzed. Scale bars: 100 μm for (**a**–**c**) and 500 μm for (**d**). *P < 0.05 by Student’s *t* test. Error bars are defined as s.d.

To investigate the role of FOXF2 in regulating various processes underlying breast cancer bone metastasis, we forced the ectopic expression of FOXF2 in MCF-7 cells and overexpressed or knocked down FOXF2 in MDA-MB-231 cells. The cancer cells with altered FOXF2 expression were evaluated in vitro for chemotactic migration, heterogeneous cell–cell adhesion, and soft agar colony formation in the MC3T3E1 cell-mimic bone microenvironment and BEAS-2B cell-mimic lung microenvironment. The results revealed that the chemotactic migration of MCF-7 and MDA-MB-231 cells toward MC3T3E1 cells (Fig. 3a), heterogeneity adhesion to MC3T3E1 cells (Fig. 3b), and anchorage-independent growth in soft agar with conditioned medium (CM) from MC3T3E1 (Fig. 3c) were significantly increased by forced expression of FOXF2 and decreased by knockdown of FOXF2. In contrast, these properties of TNBC/BLBC MDA-MB-231 cells were suppressed by FOXF2 overexpression and increased by FOXF2 knockdown in the BEAS-2B cell-mimic lung microenvironment. However, forced ectopic expression of FOXF2 did not affect these capabilities of luminal breast cancer MCF-7 cells in the BEAS-2B cell-mimic lung microenvironment (Fig. 3a–c). Since pulmonary fibroblasts and hepatic stellate cells are the most abundant stromal cell types in the lung and liver, primary human pulmonary fibroblasts (HPFs) and human hepatic stellate cells (HHSCs) were also used to mimic the lung and liver microenvironment to evaluate the lung and liver metastatic potential of the above cancer cells. We observed that FOXF2 negatively regulated the chemotactic migration, heterogeneous cell–cell adhesion, and soft agar colony formation of MDA-MB-231 cells in the HPF-mimic lung microenvironment and HHSC-mimic liver microenvironment. Surprisingly, forced ectopic expression of FOXF2 enhanced these capabilities of MCF-7 cells in the HPF-mimic lung microenvironment and HHSC-mimic liver microenvironment (Supplementary Fig. 3a–c).

Because the bone metastasis of breast cancer cells results in osteolytic lesions, we tested whether FOXF2 drives cancer cells to induce osteoclastogenesis. Pre-induced primary preosteoclasts were incubated with the CM from cancer cells treated as above. TRAP staining showed that the number and size of TRAP+ osteoclasts induced by the CM from FOXF2-overexpressing cancer cells were significantly increased compared with those induced by control cell CM. Conversely, the number and size of TRAP+ osteoclasts were reduced by the CM from FOXF2-depleted cells compared with that from control cells (Fig. 3d).

Together, these results indicate that high FOXF2 expression confers the advantages of homing to, residing in, and growing in the bone microenvironment on both luminal and BLBC cells, which promotes osteoclast formation and subsequently results in bone lesions. However, FOXF2 may play opposing roles in luminal and BLBC cells for regulating the lung and liver metastatic potential. FOXF2 controls the colonization of BLBC cells in the lung and liver microenvironment, and FOXF2 depletion enables BLBC cells to colonize the lung and liver. FOXF2 may enhance this potential of luminal cells, which is facilitated by stromal cells in the lung and liver.

### FOXF2 pleiotropically transactivates BRGs

**Fig. 4:**
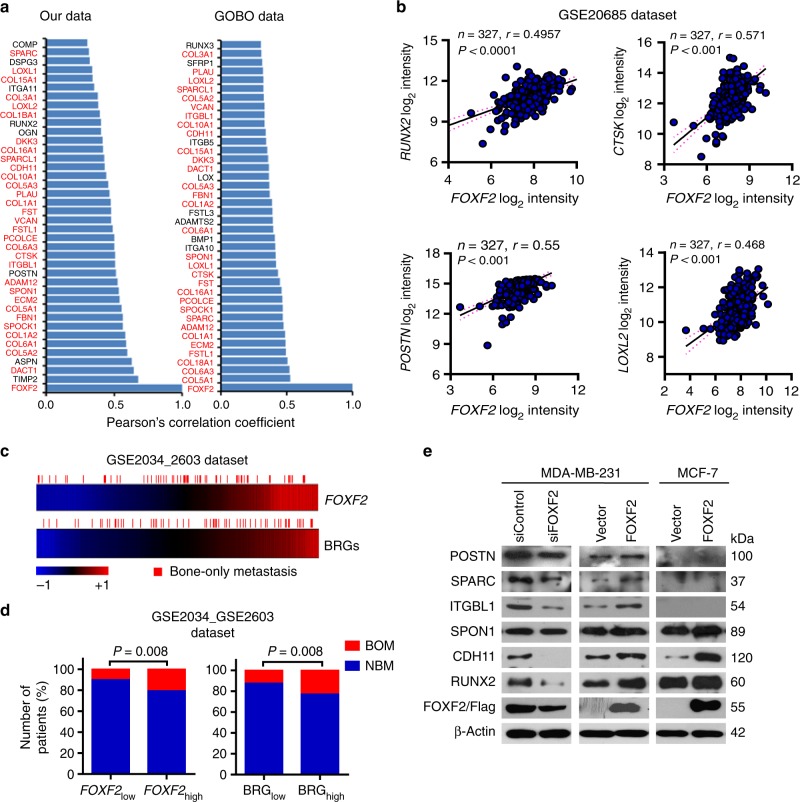
FOXF2 confers osteomimetic features on breast cancer cells and facilitates bone metastasis. **a** Genes coexpressed with FOXF2 in breast cancer were analyzed based on our gene expression profiling data set of 49 primary breast cancer tissues and the GOBO tool. The cutoff value of Pearson’s correlation coefficient was defined as 0.3. **b** The correlation of the expression of *FOXF2* with BRGs in primary breast cancer tissues based on GSE20685 data set was analyzed by Pearson’s correlation analysis. **c** Heatmaps (top) show the correlation of the frequency of bone-only metastasis (BOM) with the expression of *FOXF2* and the set of BRGs coexpressed with *FOXF2* based on the GSE2034_GSE2603 data set. The score of the set of BRGs was defined as an average of the normalized intensity of each gene in all patients. **d** The BOM and non-bone metastasis (NBM) in the *FOXF2*_high_ and *FOXF2*_low_ groups and in the *BRG*_high_ and *BRG*_low_ groups were compared. **e** The expression of proteins encoded by BRGs in the indicated cells was detected by immunoblot

To investigate the mechanism by which FOXF2 drives breast cancer bone metastasis, we screened genes that were coexpressed with *FOXF2* based on our gene expression profiling data set of 49 primary breast cancer tissues^9^ and the Gene expression-based Outcome for Breast cancer Online (GOBO, http://co.bmc.lu.se/gobo/) tool^10^. The cutoff value of Pearson’s correlation coefficient was defined as 0.3, and *P-*value was set to less than 0.05. A set of BRGs was identified as coexpression with *FOXF2* in breast cancer tissues (Fig. 4a; Supplementary Fig. 4a). The validation based on the GSE20685 data set confirmed that *FOXF2* expression positively correlated with the expression of BRGs, e.g., runt-related transcription factor 2 (*RUNX2*), periostin (*POSTN*), cathepsin K (*CTSK*), and lysyl oxidase-like 2 (*LOXL2*; Fig. 4b). These data indicate that FOXF2 is involved in the formation of the osteomimetic phenotype of breast cancer cells.

Since breast cancer cells with osteomimetic features have the advantage of osteotropism, we analyzed the relationship between the expression of *FOXF2* and BRGs in primary breast cancer tissues with bone-only metastasis based on the GSE2034_GSE2603 data set. The heatmap (Fig. 4c) shows that patients with high expression of *FOXF2* or BRGs in tumors had a higher frequency (47/181 or 34/115) of suffering bone-only metastasis than those with low expression of *FOXF2* or BRGs in tumors (14/126 or 27/192; Fig. 4d), indicating that breast cancers with high expression of *FOXF2* or coexpression of *FOXF2* and BRGs preferentially metastasize to bone.

To investigate whether FOXF2 directly regulates the set of BRGs, we analyzed the 2 -kb upstream region of the promoters for the FOXF2-binding sequence. Indeed, multiple BRGs are potentially regulated by FOXF2 (Table 1). Chromatin immunoprecipitation (ChIP) assays confirmed the binding of FOXF2 to the promoter regions of BRGs containing the candidate-binding sites in MCF-7 and/or MDA-MB-231 cells (Supplementary Fig. 4b). The detection of proteins encoded by BRGs in MCF-7 and MDA-MB-231 cells with FOXF2 overexpression and/or FOXF2 knockdown showed that FOXF2 positively regulated the protein expression of cadherin 11 (CDH11) and spondin 1 (SPON1) in both MCF-7 and MDA-MB-231 cells, as well as the protein expression of integrin subunit beta-like 1 (ITGBL1), POSTN/osteoblast-specific factor (OSF-2), secreted protein acidic and cysteine-rich (SPARC)/osteonectin (OSN), and RUNX2 in only MDA-MB-231 cells (Fig. 4e).

**Table 1:** BRGs potentially targeted by FOXF2

Gene symbol	Reference sequence number	Gene description	FOXF2-binding site related to TSS
*ITGBL1*	NM_001271756.1	Integrin, beta-like 1 isoform 3	−659/−652
			−1199/−1192
*CSPG2*	NM_001126336.2	Chondroitin sulfate proteoglycan 2/versican (VCAN)	−1422/−1415
*COMP*	NM_000095.2	Cartilage oligomeric matrix protein	−32/−25
*DSPG3*	NM_004950.4	Dermatan sulphate proteoglycan 3/epiphycan (EPYC)	−547/−540
			−1585/−1578
			−1972/−1965
*FST*	NM_006350.3	Follistatin	−1050/−1043
*OGN*	NM_014057.4	Osteoglycin	−154/−147
*POSTN*	NM_001135934.1	Periostin/osteoblast-specific factor 2 (OSF-2)	−990/−983
*SPARC*	NM_001309443.1	Secreted protein, acidic, cysteine-rich/osteonectin (OSN)	−795/−788
*SPON1*	NM_006108.3	Spondin 1	−69/−62
*CTSK*	NM_000396.3	Cathepsin K	−586/−579
			−732/−725
			−1602/−1595
*LOXL2*	NM_002318.2	Lysyl oxidase-like 2	−413/−406
*PLAU*	NM_001145031.2	Plasminogen activator/urokinase (uPA)	−1082/−1075
			−1109/−1102
			−1784/−1777

**Fig. 5:**
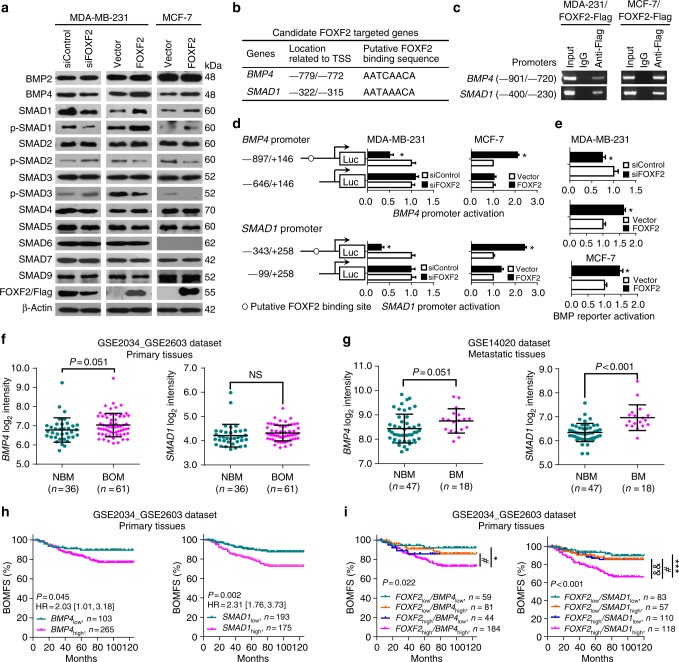
FOXF2 confers osteomimetic features and osteotropism on breast cancer cells by direct transactivation of the BMP4/SMAD1 signaling pathway. **a** The protein levels of BMPs and SMADs in the indicated cells were detected by immunoblot. **b** Candidate FOXF2 target genes in the BMP/SMAD signaling pathway are shown. **c** The binding of FOXF2 to the *BMP4* and *SMAD1* promoters containing or lacking FOXF2-binding sequences was assessed by ChIP assays. **d** *BMP4* and *SMAD1* promoter activation in the indicated cells was assessed by dual-luciferase reporter assays. pGL3-BMP4 or pGL3-SMAD1 promoter luciferase reporter constructs containing or lacking the FOXF2-binding element were transfected into the indicated cells. **e** BMP reporter activation was assessed based on the fluorescence intensity of GFP. *P < 0.05 by Student’s *t* test. Error bars are defined as s.d. **f** *BMP4* and *SMAD1* mRNA levels in primary breast cancer tissues that developed bone-only metastasis (BOM; *n* = 61) or metastases in other distant organs with or without bone metastasis (NBM; *n* = 36) were analyzed based on the GSE2034_GSE2603 data set. **g** *BMP4* and *SMAD1* mRNA levels in metastatic tissues in bone (BM; *n* = 18) and in other distant organs (NBM; *n* = 47) were analyzed based on the GSE14020 data set. **h**, **i** Kaplan–Meier analysis of the BOMFS rate of patients with different *BMP4* or *SMAD1* mRNA levels (**h**) or combined *FOXF2/BMP4* and *FOXF2*/*SMAD1* mRNA levels (**i**) in primary breast cancers was performed based on the GSE2034_GSE2603 data set. *P < 0.05 and ***P < 0.001, *FOXF2*_high_/*BMP4*_high_ vs. *FOXF2*_low_*/BMP4*_low_ and *FOXF2*_high_/*SMAD1*_high_ vs. *FOXF2*_low_/*SMAD1*_low_; ^#^*P* < 0.05, *FOXF2*_high_/*BMP4*_high_ vs. *FOXF2*_low_/*BMP4*_high_ or *FOXF2*_high_/*SMAD1*_high_ vs. *FOXF2*_low_/*SMAD1*_high_; ^&&^*P* < 0.01, *FOXF2*_high_/*SMAD1*_high_ vs. *FOXF2*_high_/*SMAD1*_low_

Although *FOXF2* was coexpressed with a set of BRGs in breast cancer, FOXF2 directly regulated some BRGs, but not others. We speculated that FOXF2 may regulate the bone morphogenetic protein (BMP)/SMAD signaling pathway that is crucial for bone differentiation and remodeling. The detection of BMPs and SMADs expression in the above cells showed that FOXF2 positively regulated BMP4, SMAD1 and phosphorylated SMAD1 (p-SMAD1), negatively regulated p-SMAD2 and p-SMAD3, and did not regulate BMP2, SMAD2, SMAD3, SMAD4, SMAD5, SMAD6, SMAD7, or SMAD9 expression (Fig. 5a). The detection of secreted proteins in the CM confirmed that FOXF2 positively regulated the secretion of BMP4 but not of BMP2 (Supplementary Fig. 5). Since *BMP4* and *SMAD1* are candidate targets of FOXF2 (Fig. 5b), we investigated the direct effect of FOXF2 on *BMP4* and *SMAD1*. ChIP assays showed that FOXF2 protein bound to the *BMP4* and *SMAD1* promoter regions containing putative FOXF2-binding sequences in both MDA-MB-231 and MCF-7 cells (Fig. 5c). Dual-luciferase reporter assays validated that FOXF2 positively regulated the activation of the *BMP4* and *SMAD1* promoters (Fig. 5d), as well as the BMP reporter (Fig. 5e) in MDA-MB-231 and MCF-7 cells. These results suggest that FOXF2 directly transactivates the expression of BMP4 and SMAD1, implying that FOXF2 programs osteomimetic phenotype in breast cancer cells by pleiotropic transactivation of BRGs and the BMP/SMAD signaling pathway.

To provide clinical evidence of the role of the FOXF2/BMP/SMAD axis in breast cancer bone metastasis, we analyzed the expression of *BMP4* and *SMAD1* in primary breast cancer tissues that developed bone-only metastasis (*n* = 61) or metastases in other distant organs with or without bone metastases (*n* = 36) based on the GSE2034_GSE2603 data set; we also analyzed the expression of these genes in metastatic tissues in bone (*n* = 18) and in other distant organs (*n* = 47) based on the GSE14020 data set. The results showed that *BMP4* was highly expressed in primary cancers that developed bone-only metastasis and metastatic cancers in bone, compared with primary cancers that developed other distant organ metastasis and metastatic cancers in other organs, respectively. *SMAD1* was highly expressed in metastatic cancers in bone, but not in primary tumors that developed bone-only metastasis (Fig. 5f, g).

We further analyzed the relevance of *BMP4* or *SMAD1* mRNA levels in primary breast cancer with bone-specific metastasis based on the GSE2034_GSE2603 data set. All cases were grouped into high and low *BMP4* expression groups (*BMP4*_high_ and *BMP4*_low_) or high and low *SMAD1* expression groups (*SMAD1*_high_ and *SMAD1*_low_) according to the optimized cutoff values, which had high sensitivity and specificity in separating all patients into distinct DMFS statuses. The results revealed that the rates of bone-only metastasis-free survival (BOMFS) in *BMP4*_high_ and *SMAD1*_high_ patients were lower than that in *BMP4*_low_ and *SMAD1*_low_ patients (Fig. 5h).

We then combined *FOXF2* expression levels with *BMP4* or *SMAD1* expression levels. The patients were grouped into subgroups of *FOXF2*_high_/*BMP4*_high_, *FOXF2*_high_/*BMP4*_low_, *FOXF2*_low_/*BMP4*_high_, and *FOXF2*_low_/*BMP4*_low_, or subgroups of *FOXF2*_high_/*SMAD1*_high_, *FOXF2*_high_/*SMAD1*_low_, *FOXF2*_low_/*SMAD1*_high_, and *FOXF2*_low_/*SMAD1*_low_. Among these subgroups, *FOXF2*_high_/*BMP4*_high_ or *FOXF2*_high_/*SMAD1*_high_ subgroup had significantly poorer BOMFS than the other subgroups (Fig. 5i), indicating that *BMP4*_high_ and *SMAD1*_high_ contribute to the *FOXF2*_high_-driven bone-specific metastasis of breast cancer. Together, these clinical data support the role of the FOXF2/BMP/SMAD axis in breast cancer bone metastasis.

### The BMP pathway mediates FOXF2-drived bone-specific metastasis

**Fig. 6:**
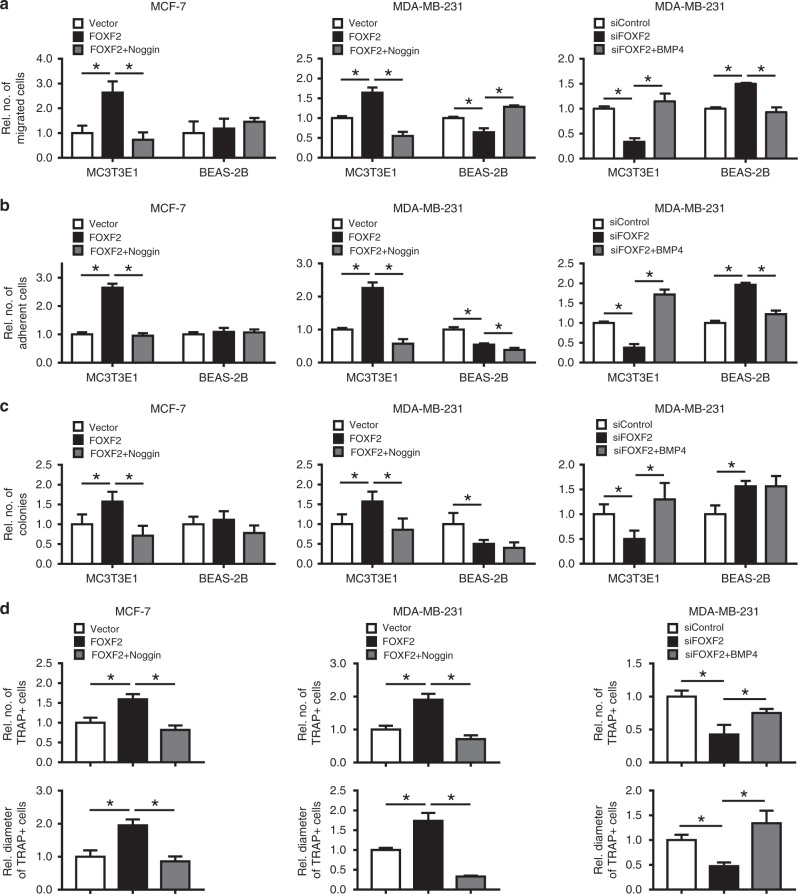
FOXF2 enhances the bone-specific metastasis of breast cancer cells through activating the BMP/SMAD signaling pathway. MCF-7 and MDA-MB-231 cells were treated as indicated. **a** Chemotactic migration of cancer cells toward MC3T3E1 or BEAS-2B cells was assessed by transwell assays. **b** Adhesion of cancer cells to MC3T3E1 or BEAS-2B cells was assessed by adding cancer cells to MC3T3E1 or BEAS-2B cells at 100% saturation, followed by incubation for 30 min. **c** Anchorage-independent growth of cancer cells in the CM from MC3T3E1 or BEAS-2B cells was assessed by soft agar colony-formation assays. **d** The ability of cancer cells for induction of osteoclast differentiation was assessed by incubation of pre-induced primary preosteoclasts with cancer cell CM containing 50 ng ml^–1^ RANKL. The number and size of mature osteoclasts with TRAP-positive multinucleated (≥3 nuclei) were analyzed. *P < 0.05 by Student’s *t* test. Error bars are defined as s.d.

To further investigate whether the BMP/SMAD signaling pathway mediates the FOXF2-regulated osteotropism of breast cancer metastasis, we added the BMP antagonist Noggin to the culture medium of MCF-7 or MDA-MB-231 cells transfected with FOXF2-expression plasmid or vector control, and added BMP4 to the culture medium of MDA-MB-231 cells transfected with small interfering RNAs (siRNAs) targeting *FOXF2* (siFOXF2) or non-targeting siRNA (siControl). Then, we assessed chemotactic migration, heterogeneous cell–cell adhesion, and soft agar colony formation of the above cancer cells in the MC3T3E1 cell-mimic bone microenvironment and the BEAS-2B cell-mimic lung microenvironment. The results revealed that Noggin attenuated FOXF2 overexpression-induced increase in the chemotactic migration of both MCF-7 and MDA-MB-231 cells toward MC3T3E1 cells (Fig. 6a), heterogeneous adhesion to MC3T3E1 cells (Fig. 6b), anchorage-independent growth in soft agar (Fig. 6c) containing MC3T3E1 CM, and osteoclast formation (Fig. 6d). Consistently, BMP4 restored the FOXF2 depletion-caused suppression of these capacities of MDA-MB-231 cells (Fig. 6a–d). However, Noggin and BMP4 did not significantly affect the FOXF2 alteration-leaded changes in these capacities of MCF-7 and MDA-MB-231 cells in the BEAS-2B cell-mimic lung microenvironment (Fig. 6a–c), HPF-mimic lung microenvironment or HHSC-mimic liver microenvironment (Supplementary Fig. 6a–c). These results indicate that the BMP/SMAD signaling pathway mediates the FOXF2-regulated osteotropism of breast cancer metastasis and osteoclastogenesis, as well as the metastatic potential of luminal cells toward the lung and liver, but is not remarkably involved in FOXF2 deficiency-conferred the lung- and liver-specific metastatic potential on BLBC cells.

**Fig. 7:**
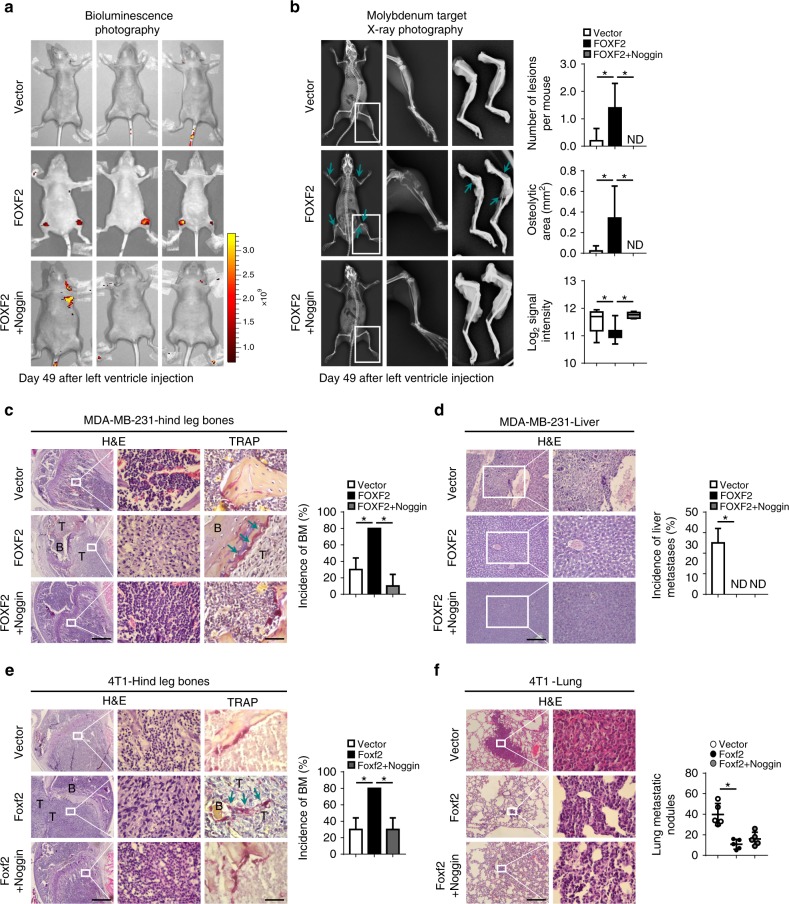
FOXF2 enhances the bone-specific metastasis of breast cancer cells through activating the BMP/SMAD signaling pathway. A total of 1 × 10^5^ MDA-MB-231 cells or 2 × 10^5^ 4T1 cells infected with LV-FOXF2/Foxf2-EGFP (FOXF2/Foxf2) or LV-EGFP vector (Vector) were injected into the left ventricle or fat pad of female nude mice (*n* = 5 per group). The mice-bearing tumors overexpressing Foxf2 were treated with 500 ng of Noggin by intraperitoneal injection three times weekly for 3 (4T1) or 4 (MDA-MB-231) weeks. **a**, **b** Bioluminescence (**a**) and X-ray imaging (**b**) of mice injected with MDA-MB-231 cells treated as indicated were analyzed. Arrows point to osteolytic lesions. **c**, **e** The metastases and osteoclasts in the hind leg bones of mice injected with MDA-MB-231 (**c**) or 4T1 (**e**) cells treated as indicated were visualized by H&E and TRAP staining, respectively. Scale bars: 500 μm for H&E staining and 50 μm for TRAP staining. Arrows point to osteolytic TRAP + osteoclasts. **d**, **f** Metastases in the liver (**d**) or lungs (**f**) of mice injected with MDA-MB-231 and 4T1 cells treated as indicated were identified by H&E staining. The metastatic nodules were counted, and the incidence of metastases was determined. Scale bars: 200 μm for (**d**) and 500 μm for (**f**). *P < 0.05 by Student’s *t* test. Error bars are defined as s.d.

To further verify the role of the BMP/SMAD signaling pathway in mediating the promotion of breast cancer bone-specific metastasis by FOXF2, we established stable MDA-MB-231 cells with FOXF2 overexpression (231-FOXF2) or vector control (231-Vector) via lentiviral infection for mouse xenograft experiments by ventricle injection. Consistent with the results generated from the fat pad injection of 4T1-Foxf2 cells (Fig. 2), FOXF2 overexpression significantly enhanced osteolytic bone metastases in limbs, as shown by bioluminescence photography (Fig. 7a) and molybdenum target X-ray photography (Fig. 7b) and validated by H&E and TRAP staining (Fig. 7c), but significantly suppressed liver metastases (Fig. 7d). As expected, the BMP antagonist Noggin significantly attenuated FOXF2-promoted bone metastasis (Fig. 7a–c), but did not affect FOXF2-suppressed visceral metastasis (Fig. 7d). The fat pad injection of 4T1-Foxf2 cells confirmed that inhibition of the BMP/SMAD signaling pathway by Noggin significantly reduced FOXF2-driven bone metastasis (Fig. 7e), but did not affect the role of FOXF2 in suppressing visceral metastasis (Fig. 7f). We also performed FOXF2 knockdown and BMP4 stimulation experiments using 231-Luc-BM cells. As respected, the results showed that FOXF2 deficiency decreased bone metastasis, and that BMP4 treatment restored bone metastases suppressed by FOXF2 depletion (Supplementary Fig. 7a–c). Further immunohistochemistry staining for pSmad1/5 in the primary tumors of 4T1/BALB/c mouse model confirmed that Foxf2 overexpression increased pSmad1/5 expression, and this effect was attenuated by Noggin treatment (Supplementary Fig. 7d). These results indicate that FOXF2 promotes breast cancer bone metastasis by directly increasing and activating the BMP/SMAD signaling pathway.

### CTSK mediates FOXF2-induced osteoclastogenesis

**Fig. 8:**
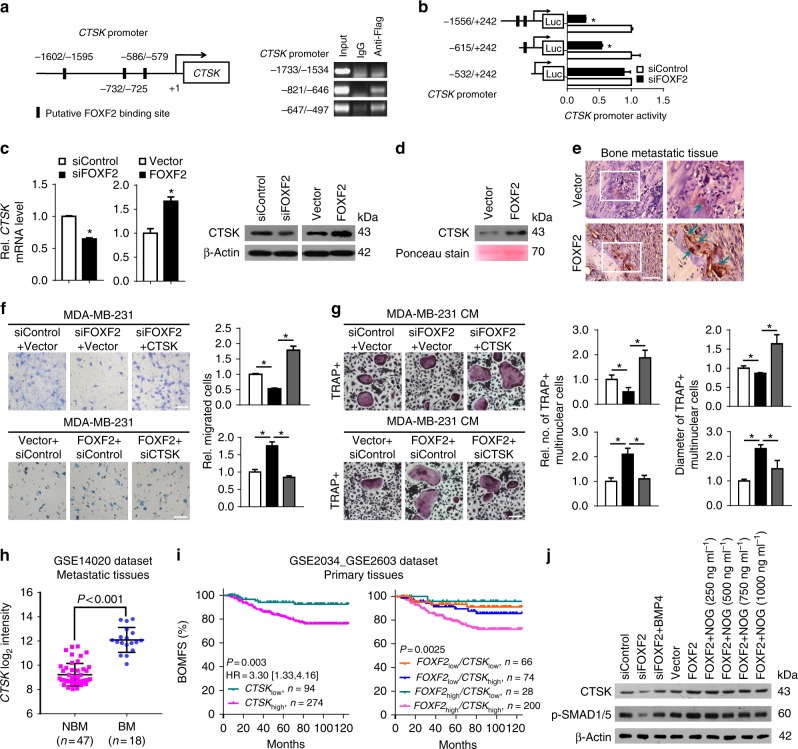
FOXF2 enhances the osteolytic bone metastasis of breast cancer cells by transactivating CTSK expression. **a** Schematic of the candidate FOXF2-binding sites in the *CTSK* promoter region (left) and the binding of FOXF2 to the *CTSK* promoter region containing the candidate-binding sites in MDA-MB-231 cells transfected with pcDNA3.1-FOXF2-Flag was determined by ChIP assays using anti-Flag antibody or IgG control (right). **b** The transcriptional activity of the *CTSK* promoter in the indicated cells was assessed by dual-luciferase reporter assays. **c** CTSK mRNA (left) and intracellular protein (right) levels in MDA-MB-231 cells, treated as indicated, were detected by RT-qPCR and immunoblot. **d** CTSK secretion levels in CM from MDA-MB-231 cells, treated as indicated, were detected by immunoblot. **e** CTSK protein expression in bone metastatic tissues in mice-bearing MDA-MB-231 cells treated as indicated was detected by immunohistochemistry. Arrows point to CTSK + cells. **f** The bone-matrix degradation and invasive capacity of the indicated cells were assessed by transwell assays. The insert was coated with bone matrix generated by MC3T3E1 cells. **g** The ability of cancer cells to induce osteoclast differentiation was assessed by incubation of pre-induced primary preosteoclasts with cancer cell CM containing 50 ng ml^–1^ RANKL. The number and size of mature TRAP-positive multinucleated (≥3 nuclei) osteoclasts were analyzed. Scale bars: 100 μm. *P < 0.05 by Student’s *t* test. Error bars are defined as s.d. **h** *CTSK* expression levels in metastatic tissues in bone (*n* = 18) and in other distant organs (*n* = 47) were compared based on the GSE14020 data set. **i** Kaplan–Meier analysis of BOMFS of patients with different *CTSK* (left) or combined *FOXF2* and *CTSK* (right) mRNA levels in primary breast cancers was performed based on the GSE2034_GSE2603 data set. **j** CTSK and phospho-SMAD1/5 protein levels in MDA-MB-231 cells, treated as indicated, were detected by immunoblot

CTSK is a well-known inducer of osteoclasts and a marker of activated osteoclasts. Because *CTSK* is coexpressed with *FOXF2* (Fig. 4a, b) and the *CTSK* proximal promoter region contains three putative *FOXF2*-binding sequences, we speculated that FOXF2 may enable breast cancer cells to induce osteoclast maturation by increasing CTSK. ChIP–PCR assays showed that FOXF2 bound to the *CTSK* proximal promoter region at −586/−579 bp and −732/−725 bp relative to the transcription start site (TSS) in MDA-MB-231 cells transfected with pcDNA3.1-FOXF2-FLAG (Fig. 8a). Dual-luciferase reporter assays revealed that FOXF2 knockdown significantly reduced the luciferase activity in MDA-MB-231 cells transfected with pGL3-CTSK containing the −732/−725 bp FOXF2-binding site compared with that in control cells (Fig. 8b). The levels of *CTSK* mRNA, intracellular protein (Fig. 8c), and secretion (Fig. 8d) were consistently regulated by FOXF2. CTSK protein expression was also increased in bone metastatic tissues formed by MDA-MB-231 cells, especially in cancer cells adjacent to osteolytic bone lesions (Fig. 8e). These results demonstrate that FOXF2 activates the transcription and expression of *CTSK* by binding to its promoter region in breast cancer cells.

To further investigate the role of the FOXF2-CTSK axis in the interaction of breast cancer cells with bone, we performed bone-matrix invasion assays to test whether FOXF2-regulated CTSK secretion by cancer cells led to the degradation and invasion of the bone matrix. MDA-MB-231 cells with FOXF2 knockdown or overexpression, FOXF2 knockdown plus adding CTSK or FOXF2 overexpression plus CTSK knockdown were seeded on transwell inserts pre-coated with bone matrix. We observed that the ability of cancer cells to invade the bone matrix was positively regulated by FOXF2 expression. The addition of CTSK reversed the suppression of bone-matrix invasion by FOXF2 knockdown, and CTSK knockdown abolished the increase in invasion due to FOXF2 overexpression (Fig. 8f). We further tested whether FOXF2-regulated CTSK secretion by cancer cells induce osteoclastogenesis. We found that the addition of CTSK to CM from FOXF2-depleted cancer cells restored the reductions in the number and size of TRAP+ osteoclasts. Conversely, CTSK knockdown attenuated the number and size of TRAP+ osteoclasts induced by the CM from FOXF2-overexpressing cancer cells (Fig. 8g). These data suggest that CTSK mediates the FOXF2-enhanced potential of osteolytic bone metastasis.

To provide clinical evidence validating the role of CTSK and the FOXF2-CTSK axis in breast cancer bone metastasis, we analyzed *CTSK* expression levels in metastatic tissues in bone and other organs based on the GSE14020 data set. The results revealed that *CTSK* was highly expressed in bone metastatic tissues compared with metastatic tissues in other distant organs (Fig. 8h). We further analyzed the clinical relevance of *CTSK* or combined *FOXF2*/*CTSK* mRNA levels with bone-specific metastasis based on the GSE2034_GSE2603 data set. The results revealed that the rate of BOMFS in *CTSK*_high_ patients is lower than that in *CTSK*_low_ patients, and *FOXF2*_high_/*CTSK*_high_ patients had a worse BOMFS than patients in the *FOXF2*_low_/*CTSK*_high_, *FOXF2*_high_/*CTSK*_low_, and *FOXF2*_low_/*CTSK*_low_ groups (Fig. 8i). These results imply that the FOXF2-CTSK axis plays a critical role in breast cancer bone-specific metastasis.

Since *CTSK* is a known target of BMP/SMAD/RUNX2^20^, we also investigated the presence of a BMP/SMAD signaling pathway-dependent mechanism for FOXF2-regulated CTSK expression by detecting CTSK and phospho-SMAD1/5 protein levels in MDA-MB-231 cells with FOXF2 knockdown plus BMP4 or with FOXF2 overexpression plus Noggin. The result revealed that FOXF2 positively regulated both CTSK and phospho-SMAD1/5 expression, and the BMP/SMAD signaling pathway mediated FOXF2-regulated CTSK expression (Fig. 8j). Thus, in addition to direct transcriptional regulation, FOXF2 also regulates CTSK expression through the BMP/SMAD signaling pathway.

## Discussion

Cancer cells that have undergone EOT are transformed into bone metastasis seeds that preferentially home to the bone microenvironment^5^, wherein they survive^6^ and colonize^7,8^. The fact that osteomimetic cancer cells commonly exist in breast cancer tissues^20^ could explain the predilection of breast cancer bone metastasis, which has an incidence as high as 80% in advanced breast cancer pants^21^. The acquisition of osteomimetic features in cancer cells is considered similar to the differentiation of the osteoblastic lineage, a process involving the progression of mesenchymal stem cells to osteoprogenitors, osteoblasts, and ultimately cortical lining cells or osteocytes^22^. The signature of BRGs ectopically coexpressed in breast cancer tissues mainly includes genes encoding matrix proteins that are expressed at the early stages of bone differentiation, e.g., collagens, SPARC/OSN, and fibronectin (FN), but does not include the matrix proteins expressed at a nonreversible differentiation stage, e.g., alkaline phosphatase (ALP), or those expressed in mature osteoblasts and terminally differentiated osteocytes, e.g., bone sialoprotein (BSP), decorin, and osteocalcin (OC)^20,22^. This evidence indicates that the fate of osteomimetic cancer cells as bone metastasis seeds is determined by their status that is similar to osteoprogenitors or pre-osteoblast-harboring stem-like properties and reversible differentiation potential. Thus, osteomimetic cancer cells could gain invasive, disseminating, and osteotropic properties, which is dependent on reciprocal interaction between cancer cells and the microenvironment during metastasis.

FOXF2 is specifically expressed in the mesenchyme adjacent to the epithelium to maintain the characteristics of mesenchymal cells and inhibit the mesenchymal transformation of epithelial cells during embryonic development and tissue differentiation^11^. Our previous studies have demonstrated that FOXF2 is highly expressed in basal-like breast cells, but less in non-basal-like breast cells, while FOXF2 deficiency promotes early-onset visceral organ metastasis of BLBC cells through triggering epithelial–mesenchymal transition-inducing transcription factor (EMT-TF) networks^12,13^. In this study, we found that FOXF2 is ectopically coexpressed with a set of BRGs in primary breast cancer tissues. Although the set of BRGs includes both collagenous and noncollagenous proteins, the target genes potentially transregulated by FOXF2 encode noncollagenous proteins primarily expressed in osteoprogenitors or pre-osteoblasts, which differs from that potentially transregulated by RUNX2. RUNX2 directly regulates the transcription of both collagenous and noncollagenous matrix proteins encoded by BRGs highly expressed in osteotropic breast cancer^20^. Importantly, FOXF2 not only directly upregulates the transcription of BRGs but also directly regulates the transcription of genes encoding key signal and effecter molecules in the osteogenic differentiation signaling pathway, BMP4 and SMAD1. The BMP signaling pathway plays a key role in breast cancer bone metastasis^23^, and this process could be regulated by zinc-finger protein 217 (ZNF217)^24^. Our study not only extends the understanding of the mechanisms responsible for the osteomimetic and osteotropic phenotypes of cancer cells but also identified a determiner of cell differentiation fate toward an osteocyte lineage. Our data also suggest that *FOXF2* mRNA level in combination with the mRNA levels of the FOXF2 target genes *BMP4*/*SMAD1* or BRGs may be biomarkers for the prediction of breast cancer bone metastasis risk, and targeting the FOXF2-BMP/SMAD axis might be a promising therapeutic strategy to manage breast cancer bone metastasis.

CTSK is produced not only by osteoclasts for osteoclast-mediated osteoclastic bone resorption^25^ but also by osteoblasts to maintain the collagenous matrix and recycle improperly processed collagen I^26^. CTSK is ectopically expressed in breast cancer cells that metastasize to bone to promote tumor cell invasiveness and contribute to bone degradation^27,28^. CTSK inhibitor therapy reduced skeletal tumor burden but did not block the growth of primary tumor, indicating that CTSK generates a favorable bone microenvironment for tumor growth by promoting bone resorption^28^. However, the mechanism regulating CTSK expression remains to be investigated. Our group has reported that *CTSK* is a direct transcriptional target of RUNX2, a master mediator of the BMP/SMAD signaling for bone development and remodeling, and that *CTSK* is coexpressed with a set of BRGs regulated by RUNX2 in primary breast cancer tissues^20^. In this study, we identified FOXF2 as another direct regulator of *CTSK* transcription and CTSK-mediated breast cancer bone metastasis. In addition to direct transcriptional regulation, FOXF2 also regulates CTSK expression through the BMP/SMAD/RUNX2 signaling pathway. Our findings suggest that the FOXF2-CTSK axis may be a potential target for the prevention and treatment of bone metastasis-caused osteolytic bone destruction.

In addition to the role of FOXF2 in promoting the bone-specific metastasis of breast cancer, we also observed a role of FOXF2 in suppressing the visceral organ metastasis of BLBC, which is consistent with our previous findings that FOXF2-deficient BLBC cells have a propensity to metastasize to visceral organs^12,13^. Recently, Zhuang et al. reported that the Wnt signaling inhibitor dickkopf (DKK1) played opposite roles in breast cancer bone and lung metastasis: promotion of bone metastasis and suppression of lung metastasis^29^. Interestingly, FOXF2 acts as an inhibitor of the Wnt signaling pathway^17,30^, which implies that the opposite roles of FOXF2 in BLBC bone and visceral organ metastasis may also involve inhibition of the Wnt signaling pathway. The subtype-specific and metastatic organ-specific patterns of FOXF2 expression and role in BLBC reflect the pleiotropic regulatory function of FOXF2 in BLBC development and metastasis.

Our previous studies indicated that FOXF2 deficiency accelerates the non-bone distant metastasis of BLBC through pleiotropic transactivation of the EMT-TFs TWIST1^12^, FOXC2^13^ and FOXQ1^31^. In this study, we observed that FOXF2 negatively regulates p-SMAD2 and p-SMAD3, indicating that FOXF2 activates the BMP/SMAD signaling pathway but inhibits the transforming frowth factor beta (TGF-β)/SMAD pathway, a key inducer of EMT/mesenchymal stem cell properties^32^. Thus, antagonism between BMP/SMAD and TGF-β/SMAD plays a critical role in the FOXF2-regulated organotropism of breast cancer metastasis. Our in vitro data suggest that ectopic expression of FOXF2 in luminal breast cancer cells not only promotes bone metastasis but also may enhance non-bone metastatic potential under conditions of stromal cell induction. However, we failed to observe any metastasis in the MCF-7-FOXF2/nude mouse ventricle injection model, and clinical data revealed that FOXF2 negatively correlated with non-bone metastasis in breast cancer cases (Supplementary Fig. 1b). In fact, TNBC/BLBC is most likely to develop visceral metastases within 3 years after diagnosis and surgery, while luminal breast cancer is more likely to metastasize to bone^33–36^. The role and mechanism of FOXF2 in regulating the non-bone distant metastasis of luminal breast cancer cells remain to be further investigated.

In summary, we provide mechanistic insights into breast cancer bone metastasis. We showed that the mesenchymal TF FOXF2, which is ectopically expressed in breast cancer cells, drives breast cancer cells to develop into bone metastasis seeds by directly programming the EOT. This study not only extends the role of FOXF2 in cancer but also proposes that the deregulation of pleiotropic TFs controlling embryonic development and tissue differentiation can lead to complex biological processes in cancer, which may underlie refractory cancer metastasis. In addition, the transactivation function of FOXF2 on BRGs implies a biological function of FOXF2 in regulating bone development and remodeling.

## Methods

### Tissue specimens

A total of 118 primary breast cancer tissues diagnosed as invasive ductal carcinoma were collected from breast cancer patients as described previously^20^. Among these cases, 15 developed bone metastasis and 18 suffered non-bone metastasis within a 10-year follow-up period after primary tumor surgery. Tumors with estrogen receptor-positive (ER+)/progesterone receptor-positive (PR+)/human epidermal growth factor receptor 2-negative (HER2−) were classified as the luminal subtype, and ER−/PR−/HER2− tumors were classified as the triple-negative subtype. The use of these specimens in this study was approved by the Institutional Review Board and the Research Ethics Committee of TMUCIH, and written consent was obtained from all participants.

### Gene expression profiling data sets

Our gene expression profiling data set of 49 primary breast cancer tissues^9^ and GOBO tool were used to identify BRGs coexpressed with *FOXF2*^10^. The GeneCards (http://www.genecards.org/) and NCBI-PubMed (http://www.ncbi.nlm.nih.gov/pubmed/) databases were used to select BRGs that encode osteoblast-specific TFs, osteoblast-specific adhesion molecules, bone-matrix proteins, bone-matrix-degrading enzymes, and growth factors that regulate bone remodeling and bone-related disease^20^. The gene expression profiling data set in Gene Expression Omnibus (GEO) under accession number GSE20685^37^ was used to validate the correlation between *FOXF2* and BRGs in primary breast cancer tissues (*n* = 327). The combined gene expression profiling data set GSE12777_GSE15026_GSE65194^38–40^ of breast cancer cell lines (*n* = 95) and the gene expression profiling data sets of primary breast cancer tissues in ArrayExpress (AE; https://www.ebi.ac.uk/arrayexpress/) under accession number E-MTAB-365^41^ (*n* = 287) and in GEO under accession number GSE3494^42^ (*n* = 118) were used to compare the difference in *FOXF2* expression levels between luminal and BLBC/ TNBC subtypes. The combined gene expression profiling data set GSE2034_GSE2603^43,44^ (*n* = 368), which contains follow-up data on organs affected by metastasis, was used to analyze the expression of *FOXF2* and BRGs in primary breast cancer tissues and the survival of patients with different affected organs based on *FOXF2* and BRG expression. Among the 119 patients suffered from distant metastasis, 83 developed bone metastasis, among which 61 developed bone-only metastasis; 39 developed lung metastasis, among which 20 developed lung-only metastasis; 15 developed liver metastasis, among which 5 developed liver-only metastasis; and 15 developed brain metastasis, among which 5 developed brain-only metastasis. The GSE14020 data set^45^ was used to compare gene expression levels in different metastatic tissues (*n* = 65), including bone metastasis (*n* = 18), lung metastasis (*n* = 20), liver metastasis (*n* = 5), and brain metastasis (*n* = 22). Optimal cutoff values of *FOXF2*, *SMAD1*, and *CTSK* mRNA levels were determined based on receiver-operating characteristic (ROC) curves and the maximum Youden’s index to separate all participants and different patient subgroups into high- and low-expression groups with distinct metastasis statuses.

### Cells and treatment

The human TNBC/BLBC cell lines MDA-MB-231 (ATCC, Manassas, VA, USA), MDA-MB-231-Luc-BM (231-Luc-BM, a subline of MDA-MB-231 cells expressing luciferase and with greater propensity for bone metastasis), and the mouse TNBC/BLBC cell line 4T1 (ATCC) were cultured in RPMI 1640 (Thermo Fisher Scientific, Carlsbad, CA, USA). The human luminal breast cancer cell line MCF-7 (ATCC) and immortalized lung epithelial cell line BEAS-2B (ATCC) were cultured in the Dulbecco’s modified Eagle’s Medium (Thermo Fisher Scientific). The mouse preosteoblastic cell line MC3T3E1 (ATCC) was cultured in Minimum Essential Medium-Alpha (Thermo Fisher Scientific). The osteoblast differentiation of MC3T3E1 cells was induced with 10 mM β-glycerol phosphate (Sigma, St. Louis, MO, USA) and 50 μg ml^−1^ L-ascorbic acid 2-phosphate (Sigma) for 14 days. Primary HPFs (ScienCell, CA, USA) and HHSCs (ScienCell) were cultured in the fibroblast medium (ScienCell) and stellate cell medium (ScienCell), respectively. All cell lines were authenticated by short tandem repeat profiling and tested for mycoplasma contamination.

Cancer cells were cocultured with MC3T3E1 cells, BEAS-2B cells, HPFs, and HHSCs or their CM diluted with three volumes of fresh normal medium^20^. Noggin (500 ng ml^−1^; PeproTech Inc, Rocky Hill, NJ, USA) or recombinant human BMP4 (20 ng ml^−1^; Sigma) was added to the culture medium to treat cancer cells for 72 h. Bovine serum albumin (for BMP4) or phosphate-buffered saline (for Noggin) was added at an equal volume in a parallel test as a control. All cell culture media were supplemented with 10% fetal bovine serum (FBS; Thermo Fisher Scientific), 100 U ml^−1^ penicillin and 100 µg ml^−1^ streptomycin, and cells were cultured at 37 °C in a humidified incubator with 5% CO_2_.

### RT-qPCR

The total RNA in tissues and cells was extracted with TRIZOL reagent (Thermo Fisher Scientific) following the manufacturer’s instructions. Reverse transcription (RT) of the total RNA was performed using SuperScript™ First-Strand Synthesis System (Thermo Fisher Scientific). Quantitative PCR (qPCR) was carried out using the Platinum® Quantitative PCR System (Thermo Fisher Scientific) or SYBR^®^ Premix Ex Taq^TM^ (TaKaRa, Dalian, China) using primers and probes listed in Supplementary Table 1. The relative expression level of the target gene was calculated by normalizing the averaged cycle threshold (Ct) values of target gene to the averaged Ct values of housekeeping gene glyceraldehyde-3-phosphate dehydrogenase (*GAPDH*; ΔCt), and determined as 2^−ΔCt^.

### Immunoblot

Cells were lysed with protein lysis buffer containing 20 mM Tris-HCl pH 7.4, 5 mM EDTA, 1% Triton-X 100, 150 mM NaCl, 1% DTT, and 1% protease inhibitor cocktail (Thermo Fisher Scientific). The proteins in the lysate were separated by SDS-PAGE and transferred to polyvinyldifluoride membranes (Bio-Rad Laboratories, Hercules, CA, USA). The membranes were blocked in 5% skimmed milk in TBST (10 mM Tris, 150 mM NaCl, 0.05% Tween 20, pH 8.3) for 1 h at room temperature, and then incubated with a primary antibody in 5% skimmed milk in TBST at 4 °C overnight. The following day, the membranes were washed in TBST and then incubated with a HRP-conjugated secondary antibody for 45 min at room temperature. The membranes were then washed in TBST, and immunoreactive protein bands were visualized by enhanced chemiluminescence reagents (Millipore, Billerica, MA, USA). The detailed information of primary and secondary antibodies is described in Supplementary Table 2.

### Immunohistochemistry

Immunohistochemistry staining of formalin-fixed and paraffin-embedded tissue specimens was carried out using a primary antibody and a peroxidase-conjugated secondary antibody with appropriate concentration. Visualization of proteins was performed with diaminobenzidine and hematoxylin was used as counterstaining. The primary antibodies were anti-phospho-SMAD1/5 (Cell Signaling Technology, cat. 9516; 1:200) and anti-CTSK (Santa Cruz, cat. 48353; 1:200).

### Plasmids and small interfering RNA transfection

Three siFOXF2 and siCTSK were synthesized by RiboBio Co. (Guangzhou, China), and the two with the best knockdown efficiency, as determined by RT-qPCR and immunoblot, were selected for transient *FOXF2* knockdown experiments. siControl was used as a control. *FOXF2* and *CTSK* cDNAs were inserted into the pcDNA3.1-HA, pcDNA3.1-Flag, or pcDNA3.1 vector. Plasmid and siRNA transfections were performed using Lipofectamine 2000 (Thermo Fisher Scientific), according to the manufacturer’s instructions.

### Lentivirus infection

Lentiviruses containing fusions of *EGFP* cDNA with human or mouse full-length *FOXF2*/*Foxf2* (LV-FOXF2/Foxf2-EGFP), short-hairpin RNA targeting *FOXF2*/*Foxf2* (LV-shFOXF2/shFoxf2-EGFP), or their controls were constructed by GeneChem Co., Ltd, Shanghai, China or Cyagen Biosciences Inc, China. MDA-MB-231 and 4T1 cells were infected with LV-FOXF2/Foxf2-EGFP, LV-shFOXF2/shFoxf2-EGFP, or their controls. The cells with stable overexpression or knockdown of FOXF2/Foxf2 were selected by puromycin and identified by RT-qPCR and immunoblot.

### Chemotactic migration assay

Chemotactic migration of breast cancer cells toward a mimic organ microenvironment was assessed using transwell inserts (8-μm pore size; BD Biosciences, CA, USA) in a 24-well plate. MC3T3E1 cells, BEAS-2B cells, HPFs, or HHSCs were pre-seeded in the lower chamber with the culture medium containing 10% FBS. Cancer cells transfected with FOXF2 plasmid, siFOXF2#1/#2, or their controls were seeded in the upper chamber, and were allowed to migrate for an appropriate time. The non-migrating cells on the upper surface of the membrane were removed by wiping. The migrating cells were fixed with 4% paraformaldehyde, stained with a Rapid Wight-Giemsa Staining Kit (BBI Life Sciences, Shanghai, China), and counted under a microscope in at least three predetermined fields for each chamber at ×200 magnification.

### Heterogeneous cell–cell adhesion assay

Cancer cells transfected with GFP-labeled FOXF2 plasmid, siFOXF2#1/#2, or their controls were seeded on pre-inoculated MC3T3E1 cells, BEAS-2B cells, HPFs, or HHSCs that were grown to nearly 100% confluence in a 24-well plate for the appropriate time. The non-adherent cancer cells were removed. The adherent cells were fixed with 4% paraformaldehyde and counted under a microscope in at least three predetermined fields for each well at ×200 magnification.

### Soft agar colony-formation assay

Cancer cells were suspended in 0.3% agarose in the CM from MC3T3E1 cells, BEAS-2B cells, HPFs, or HHSCs with 10% FBS and placed on top of the 0.6% agarose gel in the serum-free normal medium in a six-well plate. The cells were cultured for 14 days and fed twice a week with fresh CM. Cell colonies greater than 50 µm in diameter were counted.

### Osteoclastogenesis assay

Primary preosteoclasts were isolated from bone marrow cells of 6-week-old wild-type BALB/c mice and cultured overnight in the minimum Eagle’s medium with 10% FBS. Non-adherent cells were plated in a 12-well plate supplemented with 30 ng ml^–1^ macrophage colony-stimulating factor (M-CSF; PeproTech) and cultured for 3 days, and then, 50 ng ml^–1^ receptor activator of nuclear factor-κ B ligand (RANKL; PeproTech) was added and the cells were incubated for 2 days. Finally, the CM from cancer cell was added, and the cells were incubated for an additional 7 days. Multinuclear cells were stained with TRAP. The number and size of mature osteoclasts with TRAP-positive multinucleated (≥3 nuclei; TRAP+) were scored.

### Bone-matrix invasion assay

The osteoblast differentiation of MC3T3E1 cells was induced on transwell inserts. The cells were lysed by a solution containing 0.05% Triton-X-100 and 10 mM NH_4_OH, and the bone matrix remained on the insert. Cancer cells were seeded on the bone-matrix-coated insert and allowed to invade toward 10% FBS for an appropriate time. The invading cells were counted under a microscope in at least three predetermined fields for each chamber at ×200 magnification.

### ChIP–PCR assay

ChIP assay was carried out by using a ChIP Assay Kit (Millipore) according to the manufacturer’s instructions. Briefly, MCF-7 or MDA-MB-231 cells transfected with FOXF2-Flag plasmids were cross-linked with 1% formaldehyde and resuspended in lysis buffer, then sonicated and incubated with an anti-Flag antibody. The protein–DNA complexes were enriched by packed beads with protein A agarose/Salmon sperm DNA. After elution and reversal, chromosomal DNA was purified by using a GeneJET PCR Purification Kit (Thermo Fisher Scientific), according to the manufacture’s instruction. The proximal promoter regions of candidate FOXF2 target genes in the resulting DNA fragments were PCR amplified using primers listed in Supplementary Table 3.

### Dual-luciferase reporter assay

The luciferase reporters of FOXF2 target gene proximal promoter region containing the FOXF2-binding element were constructs by amplification from the normal genomic DNA using primers listed in Supplementary Table 3 and subsequently inserted into the pGL3-basic vector (Promega, Madison, WI, USA). The promoter activation of FOXF2 target genes in MCF-7 or MDA-MB-231 cells was assessed by co-transfection with luciferase reporter constructs, internal control pRL-TK and siFOXF2, FOXF2, or their control. The relative promoter activation was represented as the ratio of firefly to Renilla luciferase activity measured at 48 h post transfection using a dual-luciferase reporter assay system (Promega). The BMP-GFP Reporter plasmid (YeaSen, Shanghai, China) was used to examine activation of the BMP signaling pathway.

### Animal experiments

4T1 cells infected with LV-Foxf2-EGFP, LV-shFoxf2-EGFP, or their controls were injected into the lower right abdominal mammary fat pad of female nude mice (2 × 10^5^ cells) or BALB/c mice (5 × 10^6^ cells) aged 4–6 weeks. A total of 1 × 10^5^ MDA-MB-231 cells infected with LV-FOXF2-EGFP, LV-shFOXF2-EGFP, or control or 2 × 10^5^ 231-Luc-BM cells infected with LV-shFOXF2 or control were injected into the left ventricle of female nude mice (Nanjing Biomedical Research Institute of Nanjing University, China) aged 4–6 weeks. To inhibit or activate the BMP signaling pathway, the mice were treated with 500 ng of Noggin or 40 ng of BMP4 by intraperitoneal injection three times weekly for 4 weeks, beginning 1 (4T1) or 2 (MDA-MB-231 and 231-Luc-BM) weeks after cancer cell injection. The primary tumors in mice injected with 4T1 cells were monitored every 5 days by size measurements. Tumor volume was calculated using the formula (length × width^2^)/2. GFP bioluminescence in mice injected with MDA-MB-231 cells was imaged using a Xenogen IVIS system (Caliper Life Sciences, Hopkinton, MA, USA). The osteolytic status of the hind-leg bones and/or the whole-body skeleton was observed by molybdenum target X-ray photography. The number of osteolytic lesions, the osteolytic area, and the log_2_ signal intensity in hind-leg bones were calculated. The primary tumors, hind-leg bones, lungs, and livers were surgically harvested from the mice at killing. Bones were decalcified with 10% EDTA for 2 weeks. The metastatic nodules on the lung surface were counted, and the incidence of bone metastases and liver metastases was calculated. All tissues were fixed in 4% neutral-buffered formalin and paraffin-embedded for H&E histological staining and immunohistochemical staining. The metastasis nodules were observed visually and counted by histological examination of formalin-fixed and H&E-stained tissues. Mature osteoclasts in metastatic bone lesions were identified by TRAP staining and quantified as the number of TRAP + cells adjacent to tumor cells per millimeter interface. All animal experiments were complied with relevant ethical regulations, and protocols were approved by the Animal Ethics Committee of TMUCIH.

### Statistical analysis

Pearson’s correlation analysis was used to assess the correlation of the mRNA expression between genes based on the gene expression profiling data sets and the RT-qPCR data of breast cancer tissues. The data have been converted by logarithmic transformation and follows a normal distribution. The chi-square test or Student’s *t* test was applied to evaluate the differences in *FOXF2* mRNA expression between the luminal and basal-like/triple-negative subtypes of human breast cancer, the differences in BRG mRNA expression between metastatic tissues in bone and in other organs. Kaplan–Meier survival analysis and the log-rank test were used to compare the metastasis-free survival statuses of patients with different *FOXF2* and BRG expression levels and hazard ratios. All in vitro and in vivo assays were performed as at least two independent experiments in triplicate, and the data are presented as the mean ± standard deviation (s.d.). Student’s *t* test was used to compare the differences between the experimental group and the control group. *P* < 0.05 was considered significant.

### Reporting summary

Further information on research design is available in the Nature Research Reporting Summary linked to this article.

## Supplementary information

Supplementary Information

Reporting Summary

Source Data

## Data Availability

The gene expression profiling data sets of breast cancer tissues or cell lines that support the findings of this study are available from the corresponding references. The source data underlying all figures and supplementary figures are provided as a Source Data file.
