# Supplementary material for: FOXF2 reprograms breast cancer cells into bone metastasis seeds
Source: Nat Commun. 2019 Jun 20;10:2707. doi: 10.1038/s41467-019-10379-7 (PMC6586905; doi:10.1038/s41467-019-10379-7)
Supplement: Supplementary file 1 — Supplementary Information [file 41467_2019_10379_MOESM1_ESM.pdf]

Supplementary Information

FOXF2 reprograms breast cancer cells into bone metastasis seeds

Wang et al.

Supplementary Figures

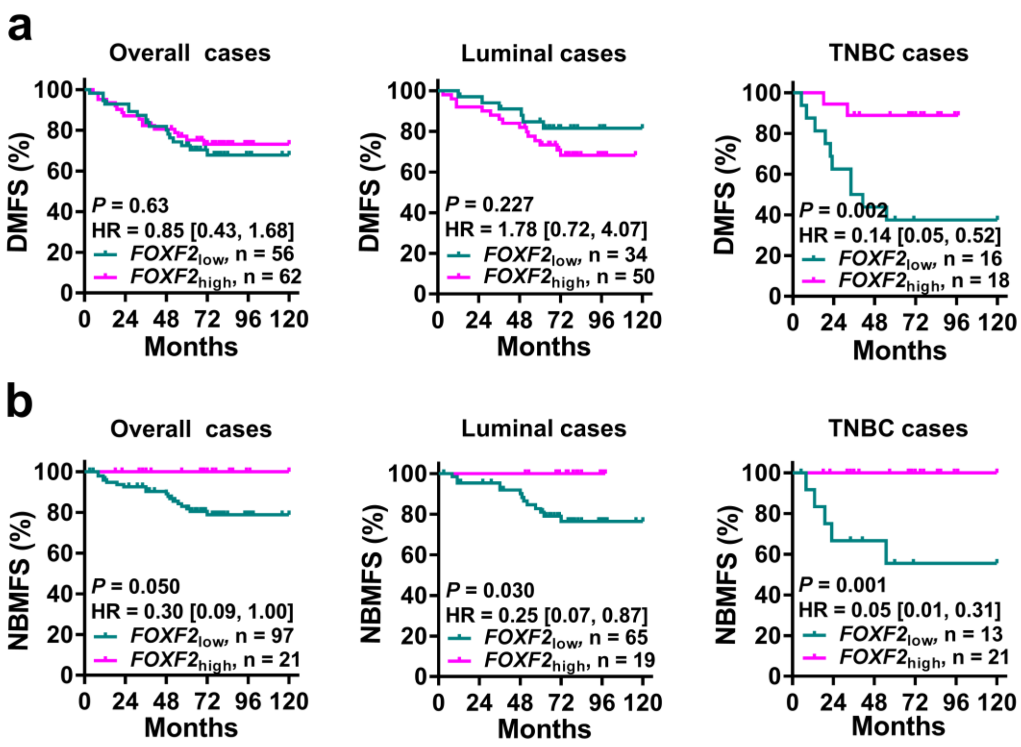

Supplementary Figure 1 | *FOXF2* expression negatively correlated with non-bone/visceral metastasis. *FOXF2* mRNA levels in primary breast cancer tissues (n = 118) were detected by RT-qPCR. The Kaplan-Meier survival curve shows the DMFS (a) and NBMFS (b) of patients in the $FOXF2_{high}$ and $FOXF2_{low}$ groups. The overall, luminal subtype and TNBC subtype cases were analyzed separately.

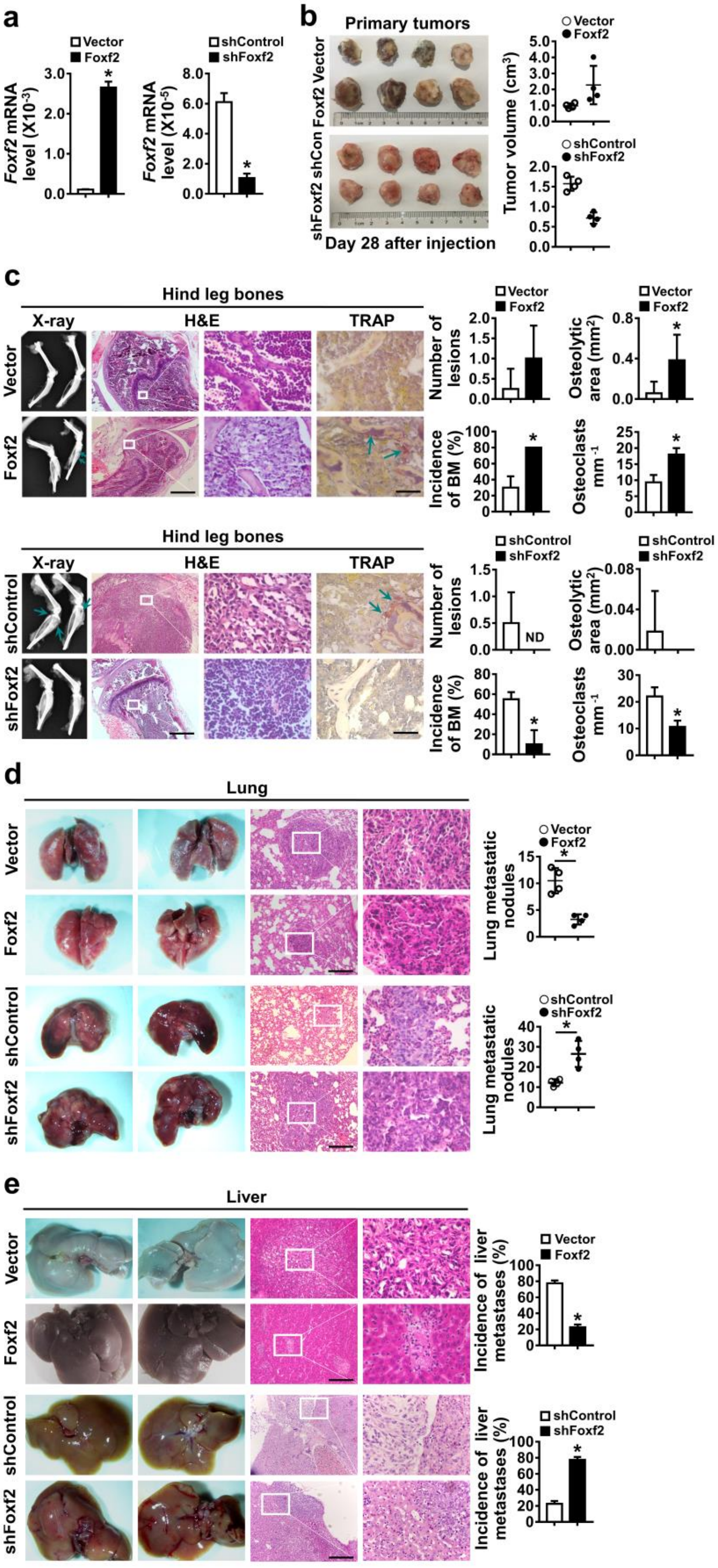

a
Vector
Foxf2
Foxf2 mRNA level (X10-3)
shControl
shFoxf2
Foxf2 mRNA level (X10-5)
b
Primary tumors
Vector
Foxf2
shCon
shFoxf2
Day 28 after injection
Tumor volume (cm3)
c
Hind leg bones
X-ray
H&E
TRAP
Number of lesions
Osteolytic area (mm2)
Incidence of BM (%)
Osteoclasts mm-1
ND
d
Lung
Lung metastatic nodules
e
Liver
Incidence of liver metastases (%)

Supplementary Figure 2 | Foxf2 deficiency suppresses the bone-specific metastasis but promotes the visceral metastasis of breast cancer cells *in vivo*. 4T1 cells were infected with LV-Foxf2-EGFP (Foxf2), LV-shFoxf2-EGFP (shFoxf2) or their controls, and 5×10^6 cells were injected into the fat pad of female BALB/c mice (n = 4 per group). The mice were killed by cervical dislocation on day 28 after cell injection. **(a)** *Foxf2* mRNA levels in the indicated cells were detected by RT-qPCR. **(b)** The volume of xenograft tumor was calculated. **(c)** The osteolytic lesions and metastases in bones were observed by X-ray, H&E staining and TRAP staining. The number of osteolytic lesions, osteolytic area and $\log_2$ signal intensity of hind leg bones detected by X-ray were calculated. Scale bars, 500 μm for H&E staining and 50 μm for TRAP staining. Arrows point to osteolytic lesions or TRAP+ cells. **(d-e)** Metastatic nodules on two sides of the lung (d) and liver (e) surface were photographed, identified by H&E staining and statistically analyzed. The metastatic nodules were counted, or the incidence of metastasis was calculated. Scale bars, 200 μm. * $P < 0.05$ by student's *t*-test. Error bars are defined as s.d.

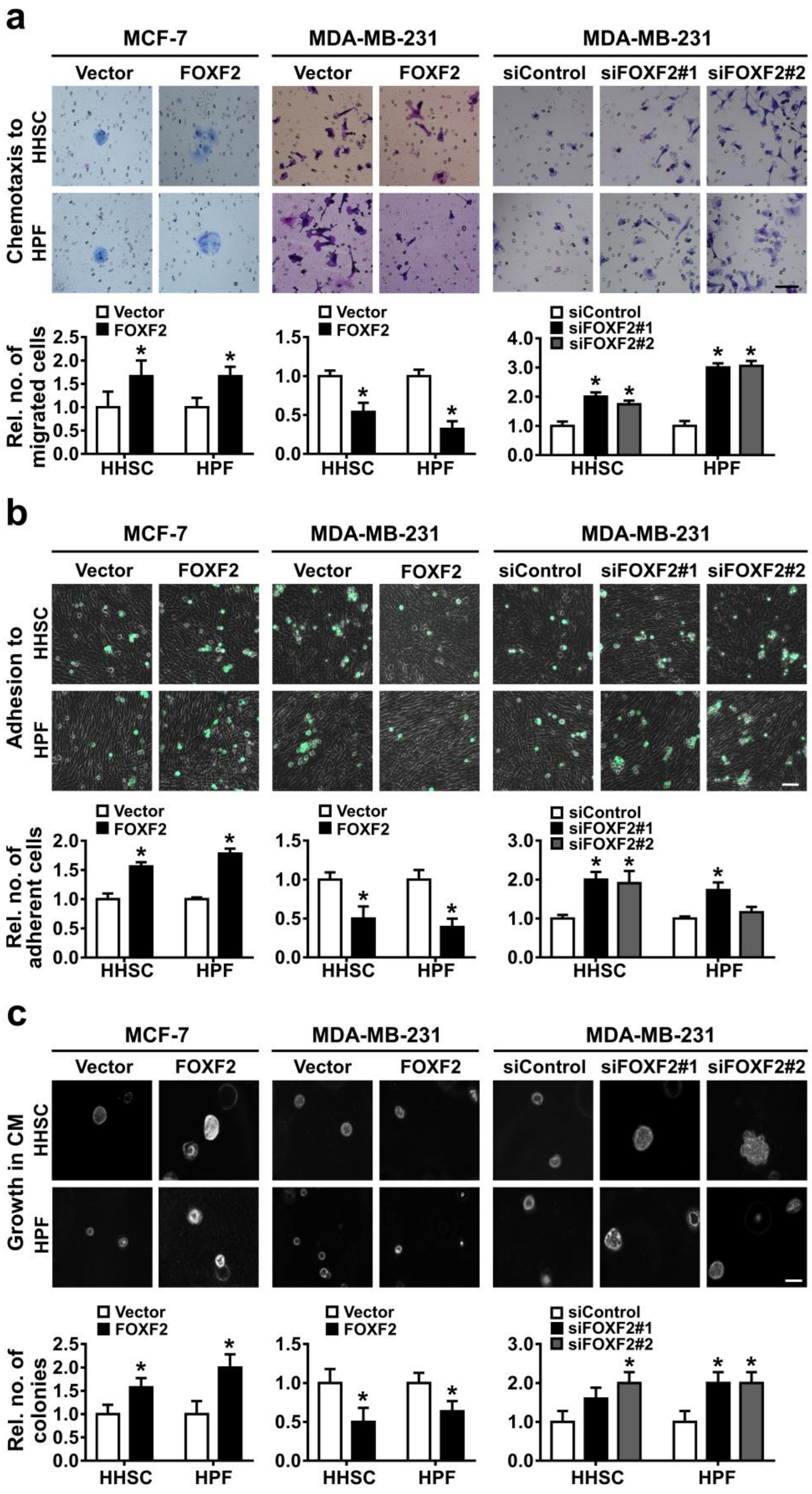
a
MCF-7
MDA-MB-231
MDA-MB-231
Vector
FOXF2
Vector
FOXF2
siControl
siFOXF2#1
siFOXF2#2
Chemotaxis to
HHSC
HPF
Vector
FOXF2
siControl
siFOXF2#1
siFOXF2#2
Rel. no. of migrated cells
HHSC
HPF
b
MCF-7
MDA-MB-231
MDA-MB-231
Vector
FOXF2
Vector
FOXF2
siControl
siFOXF2#1
siFOXF2#2
Adhesion to
HHSC
HPF
Rel. no. of adherent cells
HHSC
HPF
c
MCF-7
MDA-MB-231
MDA-MB-231
Vector
FOXF2
Vector
FOXF2
siControl
siFOXF2#1
siFOXF2#2
Growth in CM
HHSC
HPF
Rel. no. of colonies
HHSC
HPF

Supplementary Figure 3 | FOXF2 enhances the bone-specific metastasis potential of breast cancer cells. MCF-7 and MDA-MB-231 cells were treated as indicated. **(a)** Chemotactic migration of cancer cells toward HPFs or HHSCs was assessed by transwell assays. **(b)** Adhesion of cancer cells to HPFs or HHSCs was assessed by adding cancer cells on HPFs or HHSCs at 100% saturation, followed by incubation for 30 min. **(c)** Anchorage-independent growth of cancer cells in CM from HPFs or HHSCs was assessed by soft agar colony formation assays. Scale bars, 100 μm. * $P < 0.05$ by student's *t*-test. Error bars are defined as s.d.

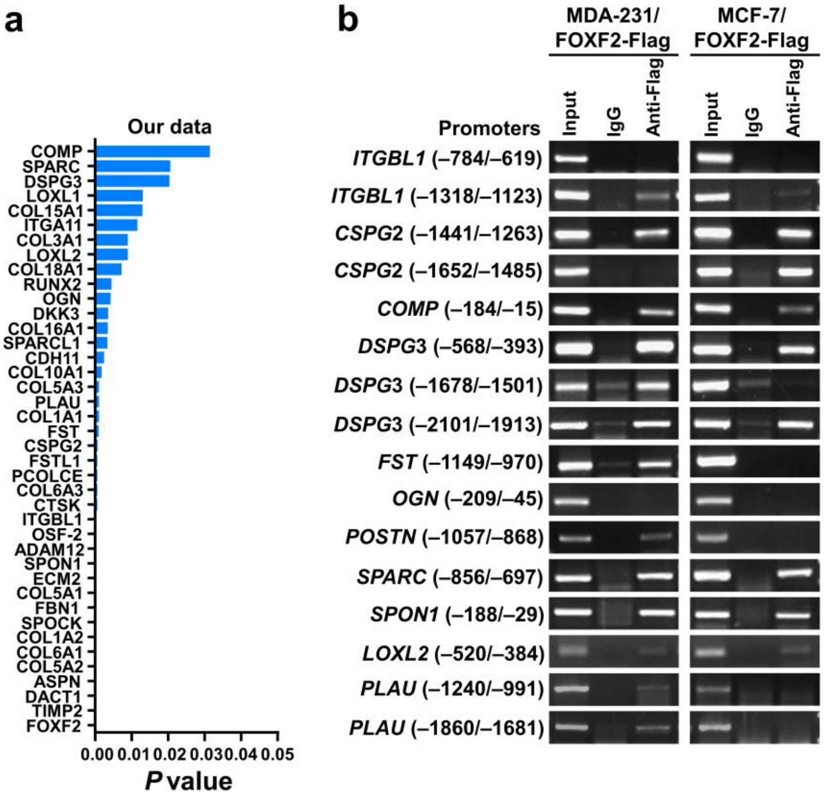

Supplementary Figure 4 | FOXF2 endows breast cancer cells with osteomimetic features that facilitate bone metastasis. (a) The *P* values of genes coexpressed with FOXF2 in primary breast cancer tissues (n = 49) were calculated by Pearson's correlation analysis. **(b)** The binding of FOXF2 to promoter regions of BRGs containing candidate binding sites in the indicated cells was determined by ChIP-PCR assay.

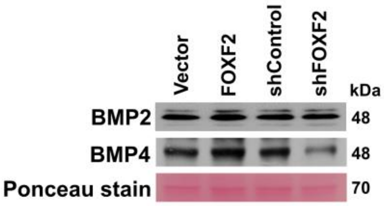

Supplementary Figure 5 | FOXF2 positively regulates BMP4 secretion in breast cancer cells. Secreted BMP2 and BMP4 protein levels in the CM from MDA-MB-231 cells treated as indicated were detected by immunoblot. Ponceau S staining served as a loading control.

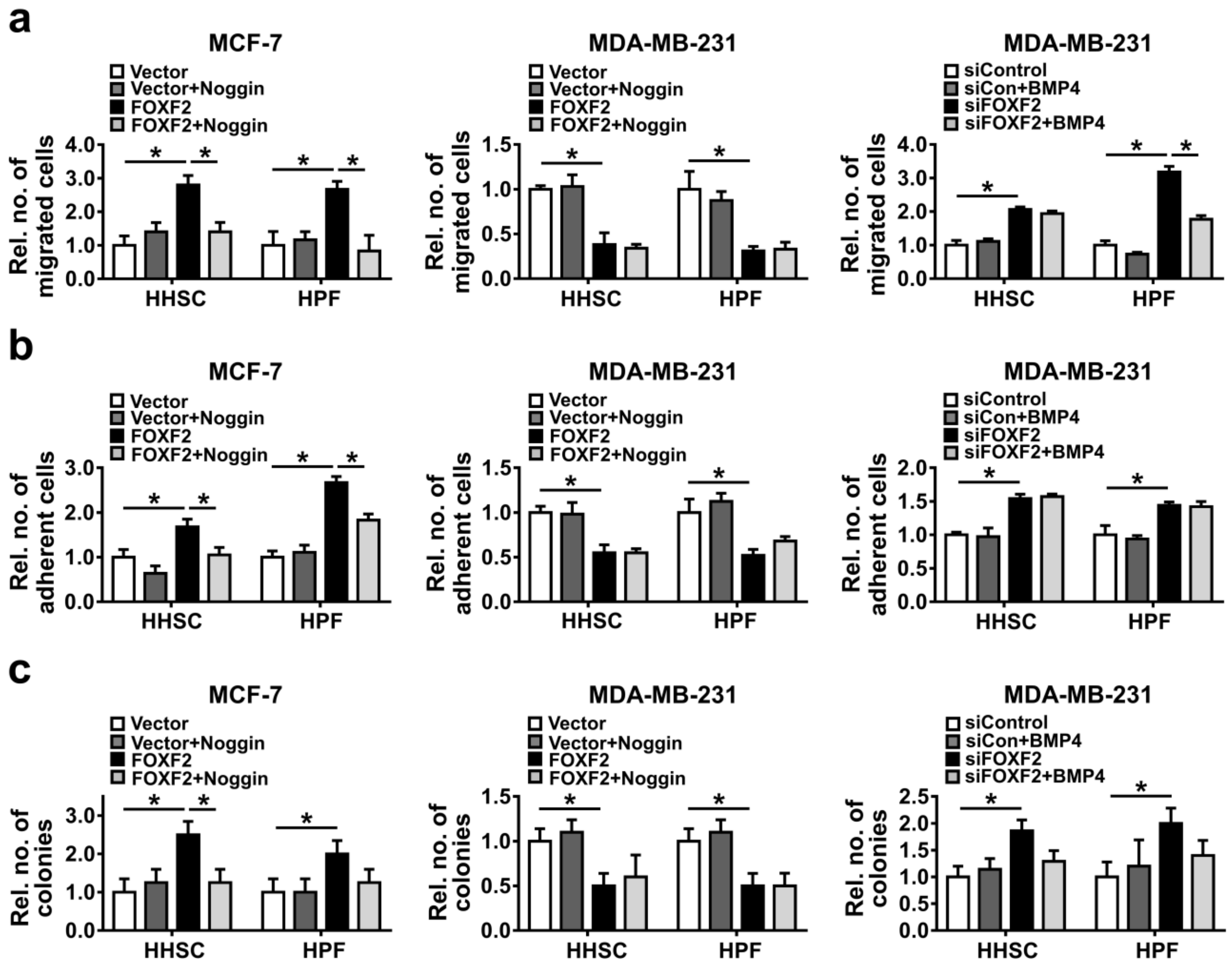

Supplementary Figure 6 | FOXF2 enhances the bone-specific metastasis of breast cancer cells through directly activating the BMP/SMAD signaling pathway. MCF-7 and MDA-MB-231 cells were treated as indicated. **(a)** Chemotactic migration of cancer cells toward HPFs or HHSCs was assessed by transwell assays. **(b)** Adhesion of cancer cells to HPFs or HHSCs was assessed by adding cancer cells to HPFs or HHSCs at 100% saturation, followed by incubation for 30 min. **(c)** Anchorage-independent growth of cancer cells in CM from HPFs or HHSCs was assessed by soft agar colony formation assays. * $P < 0.05$ by student's *t*-test. Error bars are defined as s.d.

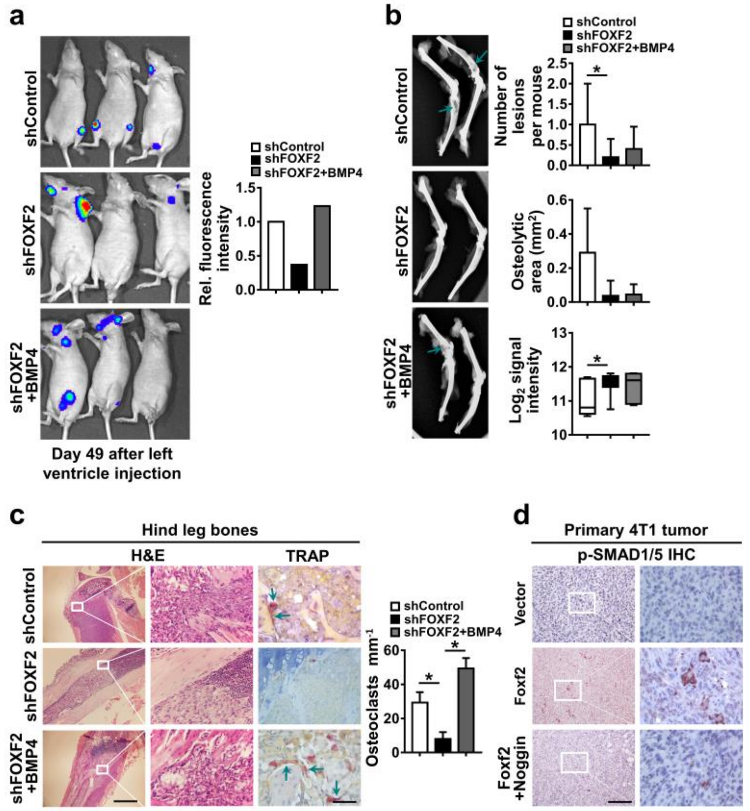

Supplementary Figure 7 | FOXF2 enhances the bone-specific metastasis of breast cancer cells through activating the BMP/SMAD signaling pathway. A total of 2 × 10^5 231-Luc-BM cells infected with LV-shFOXF2 (shFOXF2) or LV-shControl (shControl) were injected into the left ventricle of female nude mice (n = 5 per group). The mice bearing FOXF2-silenced tumors were treated with 40 ng BMP4 by intraperitoneal injection three times weekly for four weeks. **(a-b)** Bioluminescence (a) and X-ray imaging (b) of mice injected with MDA-MB-231 cells treated as indicated were analyzed. Arrows point to osteolytic lesions. **(c)** The metastases and osteoclasts in the hind leg bones of mice treated as indicated were visualized by H&E and TRAP staining. Arrows point to TRAP+ cells. **(d)** Phospho-SMAD1/5 protein expression in the indicated primary 4T1 tumors was detected by immunohistochemistry. Scale bars, 500 µm for H&E staining, 50 µm for TRAP staining and 200 µm for immunohistochemistry. * $P < 0.05$ by student's *t*-test. Error bars are defined as s.d.

Supplementary Tables

Table S1. Primers and probes for RT-qPCR assays

Genes	Primer and probe sequences	Reaction system
Gapdh	Forward: ATTGTCAGCAATGCATCCTG<br>Reverse: ATGGACTGTGGTCATGAGCC	SYBR® Premix Ex Taq™
Foxf2	Forward: CCCAGCATGTCTTCCTACTCG<br>Reverse: CGAAATCTTTCCTGTCGCACAC	
CTSK	Forward: GCAATAGCGATAATCTGAACCAT<br>Reverse: CGTTGTTCTTATTTCGAGCCAT	
GAPDH	Forward: GAAGGTGAAGGTCGGAGTC<br>Reverse: GAAGATGGTGATGGGATTTC<br>Probe: CAAGCTTCCCGTTCTCAGCC	Platinum® Quantitative PCR System
FOXF2	Forward: TGCACTCCAGCATGTCCTCCTA<br>Reverse: CGCTAGCTGAGGGATGGAAAGA<br>Probe: ACCTCTCAGTGGGACTGCCCCGTTA	

Table S2. Antibodies used in immunoblot

Name	Catalogue number	Dilution	Company
**Primary antibody**			
β-actin	A1978	1:5000	Sigma-Aldrich
SMAD1	6944	1:1000	Cell signaling technology
Phospho-SMAD1	5753	1:500	Cell signaling technology
SMAD2	3122	1:1000	Cell signaling technology
Phospho-SMAD2	8828	1:500	Cell signaling technology
SMAD3	9523	1:1000	Cell signaling technology
Phospho-SMAD3	9520	1:500	Cell signaling technology
SMAD4	9515	1:1000	Cell signaling technology
SMAD5	12534	1:1000	Cell signaling technology
SMAD6	13727	1:2000	Abcam
SMAD7	365846	1:1000	Santa Cruz
SMAD9	115900	1:1000	Abcam
Phospho-SMAD1/5	9516	1:1000	Cell signaling technology
BMP2	MAB3551	1:1000	R&D systems
BMP4	MAB757	1:1000	R&D systems
RUNX2	10758	1:1000	Santa Cruz
ITGBL1	HPA005676	1:500	Sigma-Aldrich
CDH11	4442	1:1000	Cell signaling technology
POSTN	14041	1:2000	Abcam
SPON1	40797	1:1000	Abcam
SPARC	AF941	1:1000	R&D systems
CTSK	48353	1:1000	Santa Cruz
FOXF2	H00002295-M04	1:1000	Abnova
Flag	F3165	1:2000	Sigma-Aldrich
**Secondary antibody**			
Anti-Mouse	7076	1:2000	Cell signaling technology
Anti-Rabbit	7074	1:2000	Cell signaling technology
Anti-Goat	HAF109	1:2000	R&D systems

Table S3. Primers for ChIP-PCR assays and the constructions of luciferase reporters

Genes	Region on promoters	Forward primers	Reverse primers
**Primers for ChIP-PCR assays**			
BMP4	–938/–757	AAAACAGACCAAGGGGCATC	AAGCCGTCTTAAGAACCGACA
SMAD1	–400/–230	AGGTAACTGTCAACATGCACT	CGTTCAGGCTTTCCTAACCC
CTSK	–647/–497	AAGCATTTGGAGAAGCTCAT	GACAGCACTTGAATCAATGCC
	–821/–646	ATGGAATCCAGCTAGAAACTGA	TTCCCCAACATCCAAGCAT
	–1733/–1534	ATCTGTGCTAAGCTTTACAGT	GTGCCTTATGTTTGTATGTCT
ITGBL1	–784/–619	TGTCAATCTACGGATCCAACAAT	AACACCACCTGAATCAGACCTC
	–1318/–1123	GCTCAGGCATTGTACTAAGTACCC	GGAACCTAATATTGGCATGGCTTTAAT
CSPG2	–1441/–1263	ACTGCAGAGGGCTGATAAATAACA	ACTTAGACTCCTGTACATGCGCA
	–1652/–1485	TGAAGGTTGAAGTCTGATTCTGCT	TGTTATTTATCAGCCCTCTGCAGTAAT
COMP	–184/–15	TTTCCCCACCATGTCGGATT	CCGCGGGGCCTATTTATCC
DSPG3	–568/–393	CTCCCCAAAACACGCAGATCA	AAATGTTGCAGAAACCTTACCTGTTT
	–1678/–1501	TGAGGGAAAAGGAAGAAAGTTGG	GAGTCTGTCTGAGGGATACGTG
	–2101/–1913	GGTCCTCGAGTGATTGTAACC	TTGTGTAGCATTGCAAGCAG
FST	–1149/–970	GTGACCTGCGAATGTCCAAG	GCACCGCTGAATGAAACTGA
OGN	–209/–45	AGCAGATTGTTTGATCTCCTGGGA	ACTGCTGCACTCAGTCTGCT
POSTN	–1057/–868	TGACATTACAGAGGGAGCATCT	GCATGGAGTAGTAAGAACGCAC
SPARC	–856/–697	TTTTTGAGGACAAGGACCAGGT	CAAAAAGTGGTATGAGAGGGCT
SPON1	–188/–29	CAGAGGGTCCGGAGTGTAG	TTTCCTCCGAACTGGCGAT
LOXL2	–520/–384	CCAGAGGTCACGGAATCCAA	GAAAACCGATCCTGAACCAGT
PLAU	–1240/–991	TATGTTCCTCCAAGTGTGC	CTCTGTATAGCCCATTCCA
	–1860/–1681	ATGAATCATGACGGTCCCT	CTTCAGAGCCAACCTTGCTAC
**Primers for the constructions of luciferase reporters**			
BMP4	–646/+146	GGGGTACCGGGTCTACCTCAGGGTCATCAC	CCGCTCGAGCAATCTTGAACAAACTTG CTGGA
	–897/+146	GGGGTACCTAAAAGGTTACTGCTTCTGTG	CCGCTCGAGCAATCTTGAACAAACTTG CTGGA
SMAD1	–99/+258	GGTACCATTTGGAGAGGATCCCTGGTCGCGC	CCGCTCGAGAAGTAACCCAGTCAGCA CCG
	–343/+258	GGTACCAGCGTTAATGAACATTTCAGAAAAT	CCGCTCGAGAAGTAACCCAGTCAGCA CCG
CTSK	–532/+242	GGGGTACCTCCATCAGAACTTACGGCAT	GAAGATCTGTTGCAAACGTTACCTGCT
	–607/+242	GGGGTACCAGATTGGGGATTTTAATTGAGAC	GAAGATCTGTTGCAAACGTTACCTGCT
	–1548/+242	GGGGTACCCCAGACATACAAACATAAGGCA	GAAGATCTGTTGCAAACGTTACCTGCT
